# Compartmentalization and synergy of osteoblasts drive bone formation in the regenerating fin

**DOI:** 10.1016/j.isci.2024.108841

**Published:** 2024-01-08

**Authors:** Nicole Cudak, Alejandra Cristina López-Delgado, Fabian Rost, Thomas Kurth, Mathias Lesche, Susanne Reinhardt, Andreas Dahl, Steffen Rulands, Franziska Knopf

**Affiliations:** 1 CRTD - Center for Regenerative Therapies TU Dresden, Dresden, Germany; 2 Center for Healthy Aging, Faculty of Medicine, TU Dresden, Dresden, Germany; 3 DRESDEN-concept Genome Center, DFG NGS Competence Center, c/o Center for Molecular and Cellular Bioengineering (CMCB), TU Dresden, Dresden, Germany; 4 Core Facility Electron Microscopy and Histology, Technology Platform, Center for Molecular and Cellular Bioengineering (CMCB), TU Dresden, Dresden, Germany; 5 Max Planck Institute for the Physics of Complex Systems, Dresden, Germany; 6 Ludwig-Maximilians-Universität München, Arnold-Sommerfeld-Center for Theoretical Physics, München, Germany

**Keywords:** Natural sciences, Biological sciences, Physiology, Animal physiology, Developmental biology

## Abstract

Zebrafish regenerate their fins which involves a component of cell plasticity. It is currently unclear how regenerate cells divide labor to allow for appropriate growth and patterning. Here, we studied lineage relationships of fluorescence-activated cell sorting-enriched epidermal, bone-forming (osteoblast), and (non-osteoblast) blastemal fin regenerate cells by single-cell RNA sequencing, lineage tracing, targeted osteoblast ablation, and electron microscopy. Most osteoblasts in the outgrowing regenerate derive from *osterix*+ osteoblasts, while *mmp9*+ cells reside at segment joints. Distal blastema cells contribute to distal osteoblast progenitors, suggesting compartmentalization of the regenerating appendage. Ablation of *osterix*+ osteoblasts impairs segment joint and bone matrix formation and decreases regenerate length which is partially compensated for by distal regenerate cells. Our study characterizes expression patterns and lineage relationships of rare fin regenerate cell populations, indicates inherent detection and compensation of impaired regeneration, suggests variable dependence on growth factor signaling, and demonstrates zonation of the elongating fin regenerate.

## Highlights

• Repeated fin amputation does not alter single-cell transcriptomes of regenerates
• *osterix*+ cells give rise to most bone-forming cells and impact joint formation
• Distal blastema cells are a potential source for bone regeneration
• Inhibition of Fgf signaling affects bone formation in the regenerate

## Graphical abstract

**Figure:**
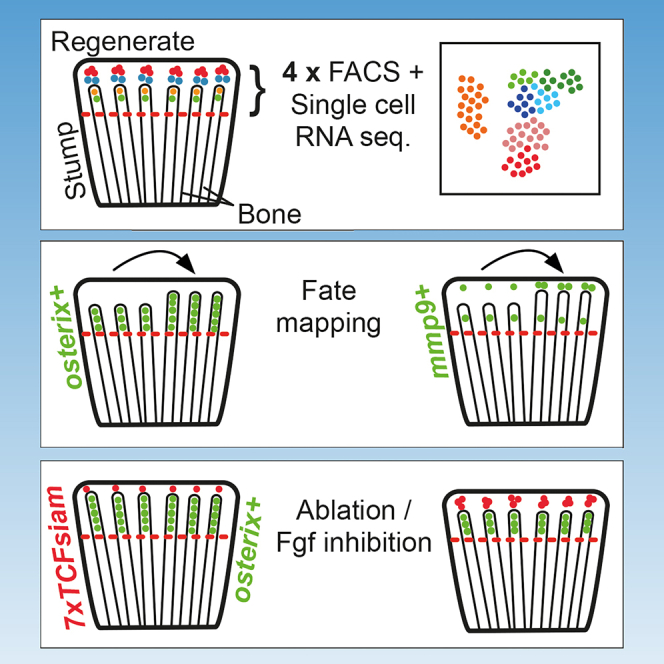

## Introduction

Zebrafish rapidly regenerate complex tissues after loss, including their appendages, the fins. Due to fast bone restoration and transparency, the fin serves as a valuable tool to study bone regeneration.^1^ After amputation, a multi-layered wound epidermis (WE) forms, which is followed by blastema formation within 2 days post amputation (dpa) and subsequent fin outgrowth.^2^ The blastema, a mass of proliferative cells accumulating at the amputation plane subdivides into different zones: a distal most blastema (DMB) of several cell diameters in size and more proximal and lateral regions (proximal blastema), in which proliferation, patterning, and osteoblast differentiation (lateral blastema domains in longitudinal section view) take place.^3^

The descendance of osteoblasts in the regenerate from stump cell populations has been intensely studied. Mature *osterix*+/*osteocalcin*+ osteoblasts in the fin stump dedifferentiate, proliferate, and migrate toward the forming blastema, where they contribute to restoration of bone matrices,^4^^,^^5^^,^^6^ although *osterix*+ cells in the fin stump are dispensable for regeneration.^7^ Other, more progenitor cell-like stump cell populations add to the osteoblast cell pool in teleost fin regenerates as well; among them are *mmp9*+ and *col10a1*+ cells at the segment joints.^8^^,^^9^ Whether and to what extent committed osteoblasts, osteoblast progenitors, and non-osteoblast cells assembled *within* the early regenerate contribute to ongoing bone formation is unclear.

In the regenerate, osteoblasts of different maturity levels reside in different locations. Osteoblast progenitors expressing *Runx2* localize to a region close to the DMB while differentiating *osterix*+ osteoblasts reside in more proximal positions. Fully mature osteoblasts expressing *osteocalcin* are found in proximity to the amputation plane at later stages of regeneration.^4^^,^^10^ Thus, based on relative position within the regenerate and marker expression, a minimum of 3 osteoblast subtypes can be distinguished in the fin regenerate, although little is known about their respective expression profiles. The aforementioned *Runx2*/*osterix* hierarchy of osteoblasts in the distal regenerate appears to be supported by a pool of distal Runx2+ cells whose existence is maintained by Wnt/β-catenin signaling and opposed by BMP (bone morphogenetic protein) signaling.^11^ While this awaits further investigation, delineation of lineage relationships between different osteoblast subtypes within the regenerate and their potential descendance from non-osteoblast sources is crucial to understand the basis of successful regeneration (with bone providing the necessary structural support to the regenerate) and to acknowledge the extent of cellular plasticity that is required for it.

In this study, we performed single-cell (sc) transcriptomics on fluorescence-activated cell sorted epidermal, "blastema" (blastema cells excluding osteoblasts), and osteoblast cell populations which may have been underrepresented in previous analyses,^12^^,^^13^ due to the high abundance of outer epidermal and proximal mesenchymal populations in fin regenerates. We identified novel subpopulations and respective marker genes and generated and tested hypotheses regarding lineage relationships of regenerate cell populations by performing trajectory inference and Cre-loxP genetic fate mapping. Tracing *osterix*+ osteoblasts and *mmp9*+ progenitor cells within the regenerate showed that both populations contribute to bone regeneration to different degrees, and that their ablation affects proliferation, patterning, and matrix formation in different parts of the regenerate. Furthermore, trajectory analysis suggested that *shha*+ cells of the basal layer of the WE (BLWE) do not contribute to the osteoblast population, while Wnt-responsive 7x*TCFsiam*+ cells (here referred to as *siam*+ cells) in the distal blastema do so, as confirmed by label-retaining cell analysis. This progenitor cell population compensates for impaired regenerate elongation after impaired regeneration due to suppression of Fgf signaling or *osterix*+ cell ablation, a recovery process that is itself independent of Fgf signaling but involves enhanced Wnt signaling. These findings, together with the identification of novel fin regenerate markers, advance our understanding of complex tissue regeneration in zebrafish.

## Results

### A robust regenerative response to repeated amputation

**Figure 1:**
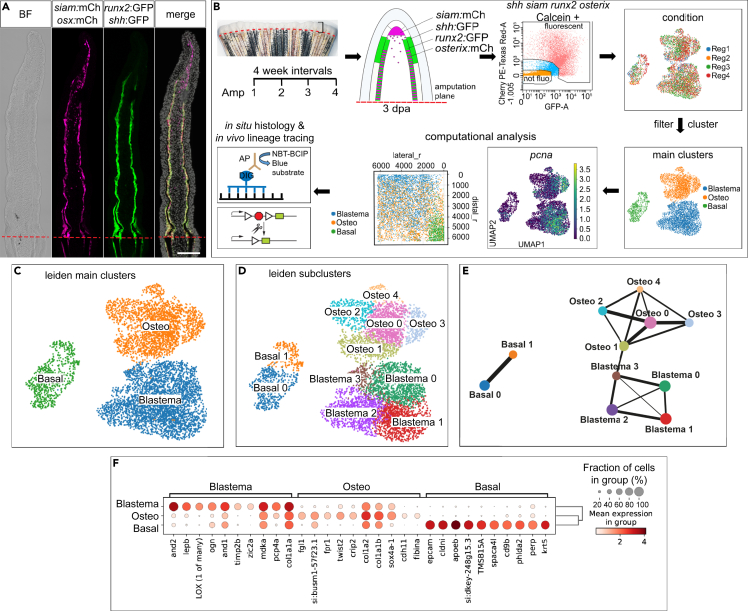
Experimental approach, the response to repeated amputation, cell clustering, and trajectory analysis (A) 3 dpa fin cryosection of quadruple transgenic reporter zebrafish. BF, bright field, mCh, mCherry, *osx*, *osterix*. Scale bar 100 μm. (B) Study design. Reg, regeneration experiment. Blastema, (non-osteoblast) blastema cells, Osteo, Osteoblasts, Basal, BLWE. Spatial reconstruction (pseudospace analysis): distal_r, pseudospace coordinate distal dimension, lateral_r, pseudospace coordinate lateral dimension. AP, alkaline phosphatase coupled antibody, DIG, digoxygenin, NBT, nitro blue tetrazolium, BCIP, 5-bromo-4-chloro-3-indolyl-phosphate. (C) Main clusters identified in the analysis. Basal, BLWE, Blastema, (non-osteoblast) blastema cells, Osteo, osteoblasts. (D) Identified subclusters. (E) PAGA analysis displaying connectivity (reflected by line thickness) between different clusters. (F) Marker gene expression in main clusters.

In order to enrich for DMB cells, osteoblasts, and cells of the lateral BLWE of the growing 3 dpa fin regenerate, we FACS (fluorescence-activated cell sorting)-isolated fluorescently labeled cells of quadruple transgenic reporter zebrafish, in which *siam*+, *Runx2*+, *osterix*+, and *shha*+ cells were labeled by either GFP or mCherry protein expression (Figures 1A, 1B, and S1A), and performed sc RNA sequencing. We repeated the procedure 4 times (Reg1-Reg4) using the same zebrafish in intervals of 4 weeks (Figures 1B and S1B). Single-cell transcriptomic analysis across samples^14^ revealed proliferating cells in distinct populations of the fin regenerate. We inferred the presence and UMAP (uniform manifold approximation and projection) location of the DMB by the near-absence of proliferating cells^3^ and confirmed the presence of proliferating cells in other populations (*pcna, mki67,* Figures 1B and S1C). Cluster composition revealed similar contributions of each amputation experiment (Figure 1B) and showed that gene expression between the first (Reg1) vs. the following (Reg2-4) samples was overall similar (Figure S1D). Significantly downregulated transcripts were only detected for 5 genes with a fold change (FC) of less than 0.5, while no significantly upregulated genes with an FC beyond 2 were detected (Figure S1E; Table S1). The overwhelming number of genes whose expression was similar between different samples (Figure S2) suggested that successive amputations do not influence gene expression levels in cells of the regenerate.

### Separation of epidermal cell clusters from osteoblast and blastema cell clusters and their relative positions in the regenerate

In order to characterize the transcriptome of cell populations of interest, we used the combined dataset of Reg1-4 with 6,668 cells for cluster analysis and identified 3 main clusters (Figure 1C) encompassing 2, 4, and 5 subclusters (Figure 1D). With the help of this dataset, we then identified marker genes for the respective subclusters and used these to localize them via RNA *in situ* hybridization (ISH). On a UMAP representation of the data, the main BLWE cluster (Basal) was clearly separated from the other two main clusters, which were connected and represented the osteoblast (Osteo) and blastema (here referring to DMB and proximal blastema cells but excluding osteoblasts which are found in lateral parts of the blastema and excluding mesenchymal cells close to the amputation plane) clusters (Figure 1D). Trajectory inference using partition-based graph abstraction (PAGA) confirmed the Osteo-Blastema cluster connection (Figure 1E),^15^ although RNA velocities visualized on the UMAP did not show a clear pattern (Figure S3A).^16^ We assessed the biological identity of the main clusters by inspecting marker genes (Figures 1F and S3B‒S3D; Table S2), which included *epcam, cldni, phlda2, fn1b, krt5*, and *lef1* in the BLWE,^12^^,^^17^^,^^18^ *and1*/2, *lepb*, *her6*, and *wnt5b* in the blastema,^19^^,^^20^^,^^21^^,^^22^ and *twist2*, *crip2*, and *cdh11* in osteoblasts^11^^,^^12^ (Figure 1F; Table S2). Gene set enrichment analysis uncovered overrepresentation of the gene ontology (GO) terms peptide metabolic process, translation, and peptide biosynthetic process in blastema cells (Table S3); ossification, skeletal system development, and extracellular matrix (ECM) organization in osteoblasts (Table S4); and cell adhesion, tight junction, and cytoskeleton in cells of the BLWE (Table S5).

**Figure 2:**
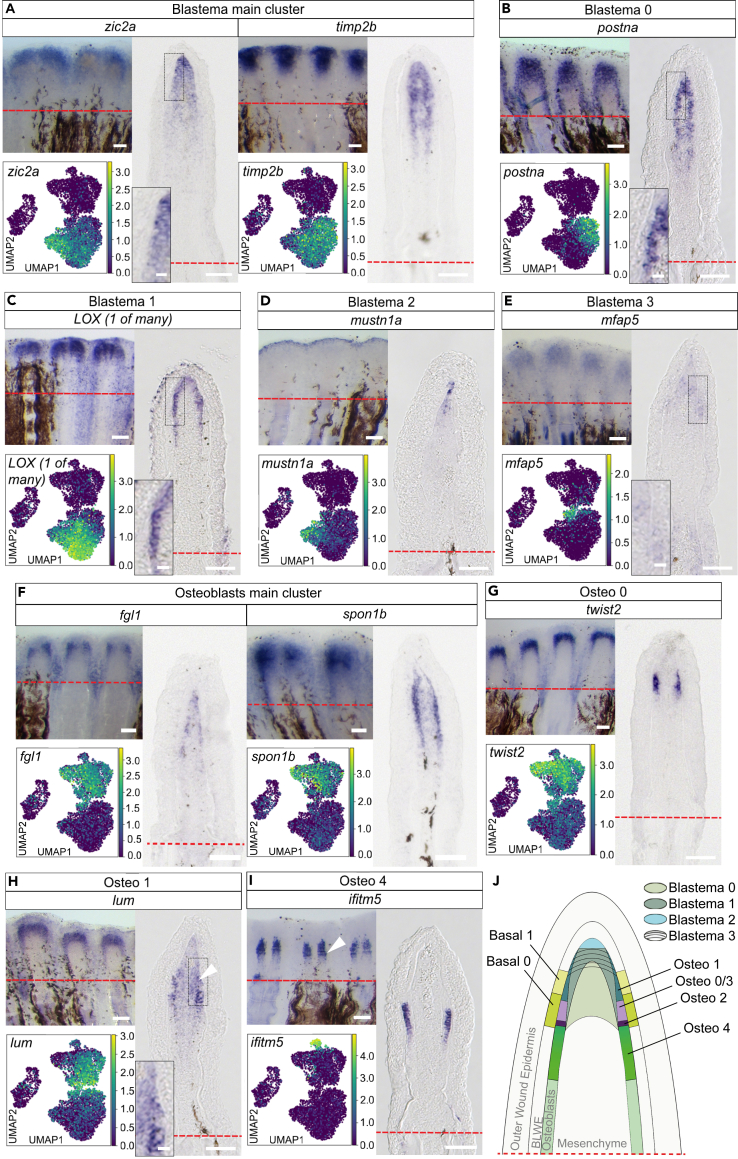
Phenotypic diversity and location of (non-osteoblast) blastema cells and osteoblasts (A) *zic2a* and *timp2b* expression. (B) *postna* in Blastema0. *postna* absence in osteoblasts and DMB. (C) *LOX (1 of many)* expression in Blastema1, touching the BLWE. (D) *mustn1a* expression. (E) *mfap5* expression in Blastema3 and some osteoblasts. (F) *fgl1* and *spon1b* expression. (G) *twist2* expression in Osteo0 cells. (H) Non-exclusive *lum* expression in Osteo1. (I) *ifitm5* expression. (J) Topology scheme of the 3 dpa regenerate, with vague distinction between Blastema0, 1, and 3. (A)–(I) UMAP, whole-mount RNA ISH (WMISH) and cryosection views. Scale bars whole mounts 100 μm, cryosections 50 μm, insets 10 μm.

Next, we studied marker gene expression in the 2 BLWE, 4 blastema, and 5 osteoblast subclusters (Figure 1D). The subclusters showed specific, although not always exclusive, thus partly overlapping, gene expression (Figures S3B‒S3D; Tables S6, S7, and S8). Subcluster “Basal0” encompassed more proximal proliferative BLWE cells expressing *col17a1b*, and *gstm.3* (Figures S3B and S3E), while more distal “Basal1” cells expressed *oclna*, *oclnb*, and *fgf24* (Figures S3B and S3E).^23^ Blastema cells, categorized into Blastema0–3 subclusters, expressed a variety of known and novel markers (Figures 1D, 2A, S3C, and S4A).^22^^,^^24^^,^^25^^,^^26^ Blastema0/1 cells represented proliferative cells positive for *postna*, *LOX*, and *tnc* (Figures 1B, 2B, 2C, S1C, S3C, and S4B; Table S6) corresponding to the highly proliferative proximal blastema.^3^^,^^19^ Blastema2 cells were characterized by high expression of *aldh1a2* (Figure S4A), *mmp13a* (Figure S4C), *fgf10a*, and *msx2b* (Figures S3D and S4A; Table S6), therefore representing more distal blastema cells,^24^^,^^25^^,^^26^^,^^27^ and included non-proliferative *pcna*− and *mki67*− cells (Figures 1B–1D and S1C) expressing high levels of *lfng*,^28^ *mustn1a*, *mmp13a*,^29^ *bmp4*,^30^ and *tgfbi* (Figures 2D, S3C, and S4C; Table S6), corresponding to the DMB. Our analyses did not show exclusive markers for this non-proliferative region which suggests that the DMB is delimited on the basis of lacking proliferation rather than exclusive marker gene expression. Blastema3 cells, located in proximity to the main osteoblast cluster in the UMAP (Figure 1D), showed high expression of *mfap5*, *pdgfrl*, and *fhl1a* (Figures 2E and S3C; Table S6).

Bone-forming cells expressed *fgl1*, *twist2*, and *spon1b* (Figures 1F, 2F, and 2G; Table S2) and comprised 5 subclusters (Figures 1D, S3D, and S4D‒S4I), characterized by often overlapping gene expression (Figures 1F and S3D). Osteo0, a central cluster with a high number of cells, was positive for *twist2, twist3*, and *tnc* expression (Figures 2G, S3D, and S4B). Osteo1, the cluster found close to Blastema3 cells in the UMAP (Figure 1D), showed *lum* and *sgk1* expression (Figures 2H and S4E). The expression of *ednrab*, *evx1*, *cx43*, *mmp9*, *hoxa13a*, and *pthlha*^17^^,^^31^^,^^32^^,^^33^ clearly defined the Osteo2 population as joint cells (Figures S3D and S4F; Table S7), while Osteo3 cells denoted proliferative osteoblasts positive for *stmn1a* (Figure S4G, compare UMAP with *pcna* and *mki67* UMAPs in Figures 1B and S1C). Osteo4 expression was characterized by high abundance of *osterix*, *bgna*, *spp1*, *col10a1a*, and *entpd5a* transcripts (Figure S4H; Table S7), which are found in differentiating zebrafish fin regenerate osteoblasts.^4^^,^^32^^,^^34^ High *mCherry* transcription (Table S7) relative to the other 4 subclusters and the expression patterns of the markers *ifitm5*, *panx3*, *sgms2*, and *fgfbp2* (Figures 2I and S4I) confirmed that Osteo4 represented differentiating osteoblasts. Next, we delineated the relative subcluster positions by comparing mRNA ISH patterns, leading to a distal regenerate topology of cells that we have enriched for (Figure 2J). The most proximal population of analyzed osteoblasts are differentiated *osterix:mCherry*+ osteoblasts labeled by *panx3*, *sgms2*, *fgfbp2a*, and *ifitm5* (Figures 2I, 2J, and S4I). Distal to this Osteo4 zone of differentiated osteoblasts (Figures 2J and S5A), occasionally within the Osteo4 zone (arrowheads in Figures 2I and S4I), Osteo2 joint cells are found (Figures S4F and S5B). Distal to the Osteo4-Osteo2 region, highly proliferative *stmn1a*+/*kpna2*+ Osteo3 cells are found (Figures 2J, S4G, and S5C), together with *twist2* and *tnc* double+ Osteo0 cells (Figure S5D). *lum*+, *sgk1*+, (Figures 2H and S4E), *abi3bpb*+, and *mxra8b*+ (Figure S4D) Osteo1 cells represent the distalmost osteoblast population within the regenerate.

Blastema2 cells encompass DMB cells. Underneath, proliferative peripheral (touching the BLWE) Blastema1 cells enriched for *LOX (1 of many)* (Figure 2C) and more central (devoid of contact with BLWE) *postna*+ Blastema0 cells (Figure 2B) can be found. *mfap5*+ Blastema3 cells (Figure 2E) reside in a position directly adjacent (distal) to Osteo1 osteoblasts (Figure 2J). Both populations share expression of *abi3bpb* and *mxra8b* (Figure S4D) and might thus represent a transition zone of blastema cells and osteoblasts. The diversity of osteoblast, blastemal, and BLWE clusters in our dataset illustrates the advantage of FACS enriching for rare cell populations of interest.

### Lineage tracing of *osterix*+ osteoblasts and *mmp9*+ cells suggests variable contribution to bone formation and the presence of different regenerate domains

**Figure 3:**
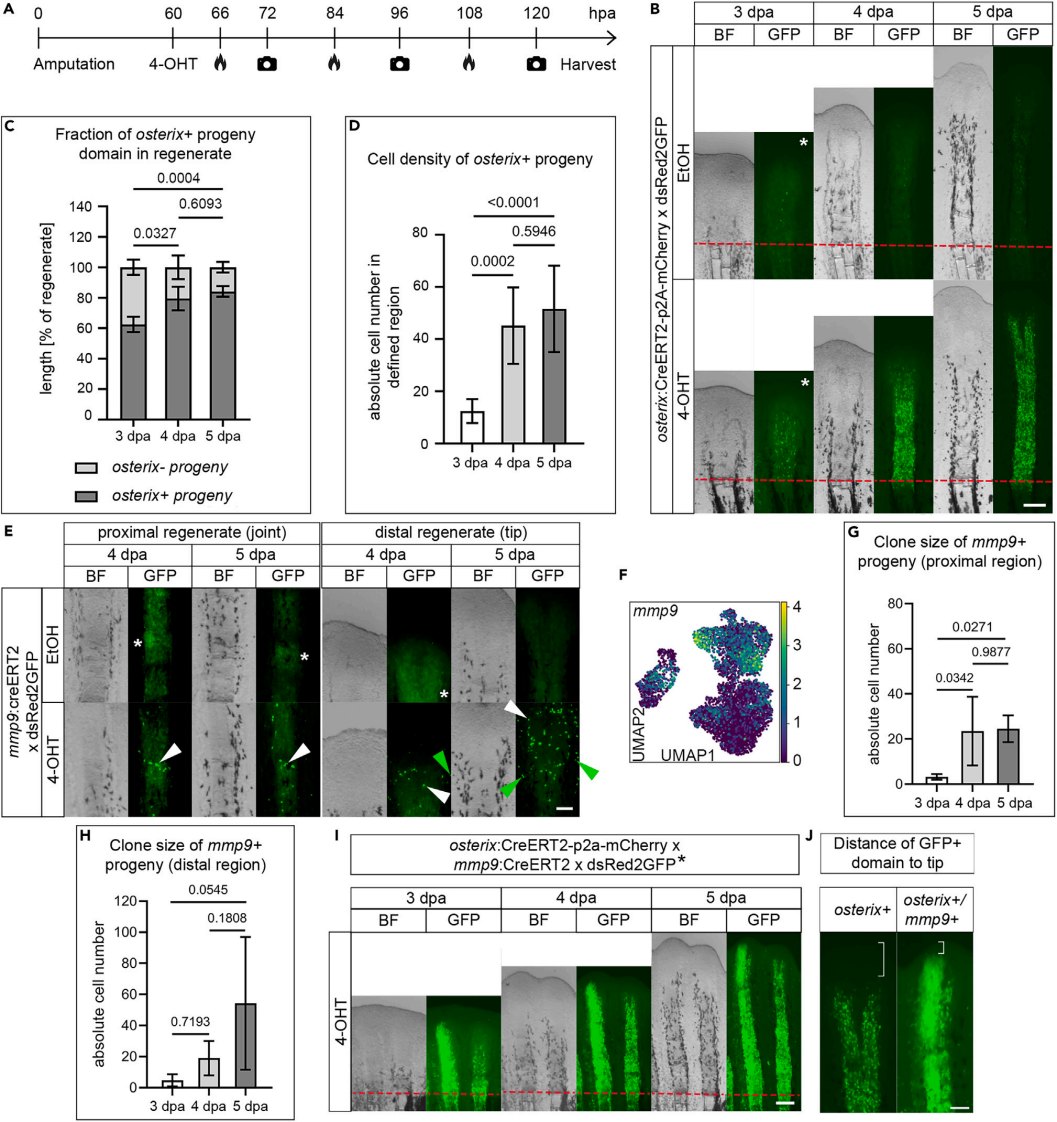
Lineage tracing of *osterix*+ and *mmp9*+ osteoblasts (A) Experimental design. 4-OHT, 4-hydroxytamoxifen. Flame icon, heat induction. Camera icon, imaging. (B) Lineage tracing of *osterix*+ cells. Asterisk, brightness and contrast increased to reveal GFP expression. Scale bar 200 μm. (C) Fraction of *osterix*+ and *osterix−* progeny at 3, 4, and 5 dpa. Kruskal-Wallis. (D) Density of *osterix*+ progeny in the 3, 4, and 5 dpa regenerate in a defined square region of interest (fin ray regenerate region close to amputation plane) of 0.028 mm^2^ (167 μm × 167 μm). One-way Anova (Tukey). (E) Lineage tracing of *mmp9*+ cells (joint cells in proximal regenerate [arrowhead], left panel, and cells in distal regenerate, right panel). Scale bar 100 μm. Asterisks indicate autofluorescence. White arrowheads, nGFP+ cells within fin ray region, green arrowheads, nGFP+ cells in interray. (F) UMAP view of *mmp9* expression. Expression is not restricted to joint cells. (G) Clone size of *mmp9*+ progeny in the proximal region of the regenerate. One-way Anova (Tukey). (H) Variable clone size of *mmp9*+ progeny in the distal region of the regenerate. One-way Anova (Tukey). (I) Lineage tracing of *osterix*+ and *mmp9*+ cells. dsRed2GFP∗, *hsp70L*:R2nlsGFP x *Actb*:dsRed2GFP. Scale bar 200 μm. (J) Magnified view of *osterix*+ progeny shown in (B) and *osterix*+/*mmp9*+ progeny shown in (I). Brackets, distance to the tip of the regenerate. Scale bar 100 μm. (B), (E) EtOH, ethanol vehicle control. (B), (I) Red dashed line, amputation plane. (B), (E), (I) BF, bright field. (C), (D), (G), (H) Mean ± SD.

Next, we made use of CreERT2-loxP-mediated lineage tracing (Figure 3A) to determine the contribution of differentiated *osterix*+ osteoblasts (including Osteo4 osteoblasts, Figures 3B–3D)^4^ and *mmp9*+ cells (including Osteo2 and likely Osteo1 osteoblasts, Figures 3E–3H, but also epidermal cells^8^ and some non-osteoblast blastema cells; Figure 3F) as the fate and extent of progeny formation of these osteoblast populations *within* the elongating regenerate were unexplored. We activated CreERT2 in *osterix*:CreERT2-p2a-mCherry x *hsp70L*:R2nlsGFP,^35^ *mmp9*:CreERT2 x *hsp70L*:R2nlsGFP, and *osterix*:CreERT2-p2a-mCherry x *mmp9*:CreERT2 x *hsp70L*:R2nlsGFP x *Actb*:dsRed2GFP^36^ zebrafish fin regenerates by injection of 4-hydroxytamoxifen (4-OHT) at 2.5 dpa, when CreERT2 is expressed in osteoblasts/osteoblast progenitors of the regenerate^4^^,^^8^ (Figure 3A). The *hsp70L*:R2nlsGFP responder line drives expression of nuclear GFP (nGFP) in cells after CreERT2-induced deletion of a floxed dsRed2 cassette (R2: *loxP* dsRed2 *loxP*) reliably after heat shock (Figure S6A)^4^; the *Actb*:dsRed2GFP responder line drives expression of GFP.^36^ *osterix*+ recombined, nGFP+ cells in *osterix*:CreERT2-p2a-mCherry x *hsp70L*:R2nlsGFP zebrafish were dispersed in the 3 dpa regenerate, their location spanning the region of Osteo4 cells (Figure 3B) and more proximal osteoblasts, in which CreERT2 and mCherry proteins are produced in the transgenic Cre-driver zebrafish (Figure S6B). At 4 and 5 dpa the regenerate had considerably and progressively enlarged, as did the nGFP+ domain (62.45% ± 5% of the regenerate at 3 dpa, 79.46% ± 7.8% at 4 dpa, and 84.18% ± 3.5% at 5 dpa, Figure 3C). At this time, the distalmost nGFP-labeled cells in the regenerate were detected ∼250 μm apart from the regenerate tip (compared to ∼350 μm at 3 dpa), with the density of nGFP-labeled regenerate cells remaining constantly high (Figure 3D). This suggests profound contribution of *osterix*+ osteoblasts and their progeny to bone-forming cells in proximal regions of the regenerate, sparing the most distal 250 μm of the regenerate. In *mmp9*:CreERT2 x *hsp70L*:R2nlsGFP zebrafish, the progeny of recombined *mmp9*+ cells labeled by nGFP was found close to the joints but also represented scattered cells within the fin rays and sometimes interrays^8^ (Figure 3E). While recombined cells at the joints neither drastically changed their position (arrowheads in Figure 3E) nor changed their number (average clone size 24 ± 10 cells, Figure 3G) until 5 dpa, distal scattered cell clones amplified in size (Figure 3E), varying considerably in number (average clone size 54 ± 42 cells, Figure 3H). This indicates a limited contribution of *mmp9*+ cell progeny at the prospective joints to the osteoblast regenerate populations, while distal *mmp9*+ cells may contribute to considerably more (pre-) osteoblasts, but also non-osteoblast tissue in the regenerate, likely derived from *mmp9*+ cells in the epidermis^8^ as well as other *mmp9*+ cell populations. In *osterix*:CreERT2-p2a-mCherry x *mmp9*:CreERT2 x *hsp70L*:R2nlsGFP x *Actb*:dsRed2GFP zebrafish, in which both *osterix*+ and *mmp9*+ cells had been recombined, more (distal) cells were labeled by GFP than in the individual Cre-driver line experiments. This was evident by a larger domain of GFP+ cells in individual fin rays of zebrafish in which both Cre-drivers were present (Figure 3I) and a concomitant shorter distance of labeled cells to the regenerate tip (brackets in Figure 3J). These data indicate that *osterix*+ cells including Osteo4 cells and *mmp9*+ cells including Osteo2 and Osteo1 cells contribute to different sets of fin regenerate osteoblasts in different compartments and at different quantities.

### Mixing of blastema and osteoblast cell populations in the distal regenerate suggests contribution of distal blastema cells to bone formation

**Figure 4:**
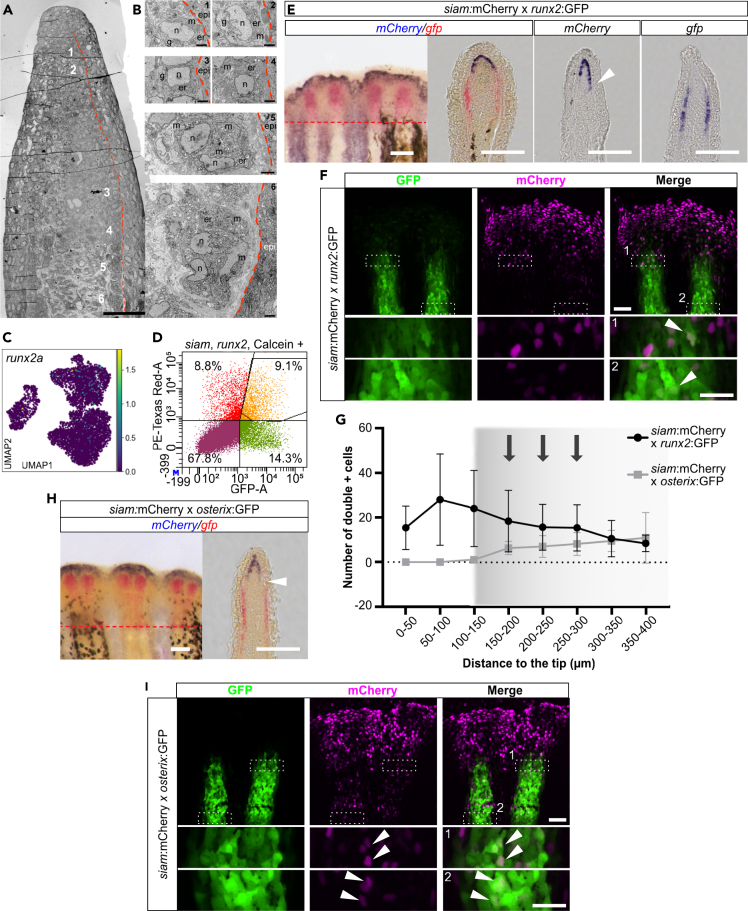
Mixing of distal blastema cells and osteoblasts (A) Overview of TEM of regenerate with positions of cells of interest indicated (1–6). Red dashed line, subepithelial basal lamina. (B) Cells underlying the BLWE with gradual increase of endoplasmic reticulum (er) from distal (1,2) via intermediate (3,4) to proximal positions. More dilated ER in proximal regions with more Golgi complexes (g) and mitochondria (m) suggesting massive protein synthesis. See also Figure S7. epi, epidermis. n, nucleus. Scale bar (A) 50 μm, (B) 2 μm. (C) UMAP of *runx2a*. (D) FACS gates to identify *siam*:mCherry+, *osterix*:GFP+, and mCherry/GFP double+ cells at 3 dpa with respective percentages of unlabeled and labeled cell populations. (E) Single and double ISH of *mCherry* and *gfp* transcripts in *siam*:mCherry x *runx2*:GFP transgenic zebrafish. Arrowhead, proximal *mCherry* expression. Red dashed line, amputation plane. Scale bars 100 μm. (F) Detection of mCherry/GFP protein double+ cells (arrowheads in boxed areas) in 3 dpa fin regenerates of *siam*:mCherry x *runx2*:GFP transgenic zebrafish. Scale bar overview 50 μm. Scale bar inset 25 μm. (G) Quantification of mCherry, GFP double+ cells in *siam*:mCherry x *runx2*:GFP, and *siam*:mCherry x *osterix*:GFP transgenic zebrafish (experiments shown in [F], [I]). Shadowed interval with arrows, regions outside of transcript detection. (H) Double ISH of *mCherry* and *gfp* transcripts in *siam*:mCherry x *osterix*:GFP transgenic zebrafish. Arrowhead, transcript negative region. Red dashed line, amputation plane. Scale bars 100 μm. (I) Detection of mCherry/GFP protein double+ cells in 3 dpa *siam*:mCherry x *osterix*:GFP fin regenerates (arrowheads in boxed areas). Scale bar overview 50 μm. Scale bar inset 25 μm. (G) Mean ± SD.

The fact that the progeny of *osterix*+ lineage-traced cells spared a ∼250 μm region at the regenerate tip indicated that other cells might contribute to distal osteoblast populations. In order to explore this possibility, we performed transmission electron microscopy (TEM) of 3 dpa regenerating fins and compared cell phenotypes of WE-underlying blastema and osteoblast cells in different proximodistal positions of the regenerate (Figures 4A, 4B, and S7). While DMB cells were small with few and flat endoplasmatic reticulum (ER) cisternae and few mitochondria and produced a thin ECM layer, putative pre-osteoblasts and osteoblasts produced more ECM and showed a more secretory phenotype with more dilated ER cisternae and more Golgi complexes and mitochondria, especially close to the amputation plane (Figures 4B and S7). We did not discern any abrupt phenotype changes between cells but rather a gradual change in morphology and tested for cells transitioning between the distal and proximal blastema. A prime candidate for a transitioning population was the *runx2*:GFP+ cell population (Figure 1A),^10^^,^^11^ in addition to distal *mmp9*+ cells in the blastema. Of note, *runx2a* transcripts were detected particularly in proliferating osteoblasts (Osteo0, Osteo3) (Figure 4C), while its expression was low in Osteo4 cells. We tested whether distal *runx2*:GFP+ pre-osteoblasts are generated by blastema cells using transgenic *siam*:mCherry x *runx2*:GFP zebrafish, in which DMB cells and pre-osteoblasts are labeled by *mCherry* and *gfp* transcripts and proteins, respectively (Figures 4D–4F). Live imaging, FACS, and combined RNA ISH and immunohistochemistry (ISH-IHC) were used to follow the respective cell progeny. Double RNA ISH showed that *gfp* and *mCherry* reporter transcript domains were sometimes clearly separated but often continuous with a slight overlap (arrowhead in Figure 4E), as previously described.^27^ ISH-IHC revealed that *siam*:mCherry protein distribution extended much more proximally than *mCherry* transcripts, and that there was only a minimum overlap between *mCherry* mRNA and GFP protein (arrowheads in Figure S8A). We hypothesized that some of these *siam*:mCherry protein+ cells had lost *siam* promoter activity and accordingly *mCherry* transcription, exited the distal Wnt-active domain, and relocated proximally to contribute to the (pre-) osteoblast cell pool. In order to test this, we performed FACS analysis in *runx2*:GFP x *siam*:mCherry fin regenerates and detected a considerable number of cells with simultaneous red and green fluorescence, i.e., mCherry and GFP protein overlap (Figure 4D). Confocal imaging of 3 dpa regenerates of the same zebrafish line revealed that GFP and mCherry double+ cells were detectable beyond 150 μm proximal to the distalmost DMB mCherry+ cells (protein level, Figures 4F and 4G). We never detected *mCherry* transcripts in such proximal locations and concluded that *siam*:mCherry+ blastema cells (potentially reflecting *and1*+, *and2*+ actinotrichia-forming cells, Figure S8B)^21^ contribute to the pool of pre-osteoblasts in the regenerate. We also detected some cells with low GFP protein levels outside of the domain of *gfp* transcription in the tip region (0–150 μm, Figure S8C), suggesting that *runx2*:GFP+ pre-osteoblasts contribute to or mix with the distal blastema cell population. We next tested whether there was any overlap between *siam*:mCherry transcripts and *gfp* transcripts in *osterix*:GFP x *siam*:mCherry zebrafish using the same approach, with *osterix*:GFP+ cells being more proximally located than *runx2*:GFP+ cells. *gfp* and *mCherry* transcript domains were clearly separated (Figure 4H). Nevertheless, GFP and mCherry protein double+ cells were detected proximal to *siam*:mCherry transcript expression (Figures 4G–4I and S8A). These observations support the hypothesis that distal Wnt-active cells contribute to the osteoblast cell pool in the distal regenerate; however, genetic fate mapping of DMB cells will be required to confirm this.

### Fgf signaling controls osteoblast and distal blastema compartment sizes

**Figure 5:**
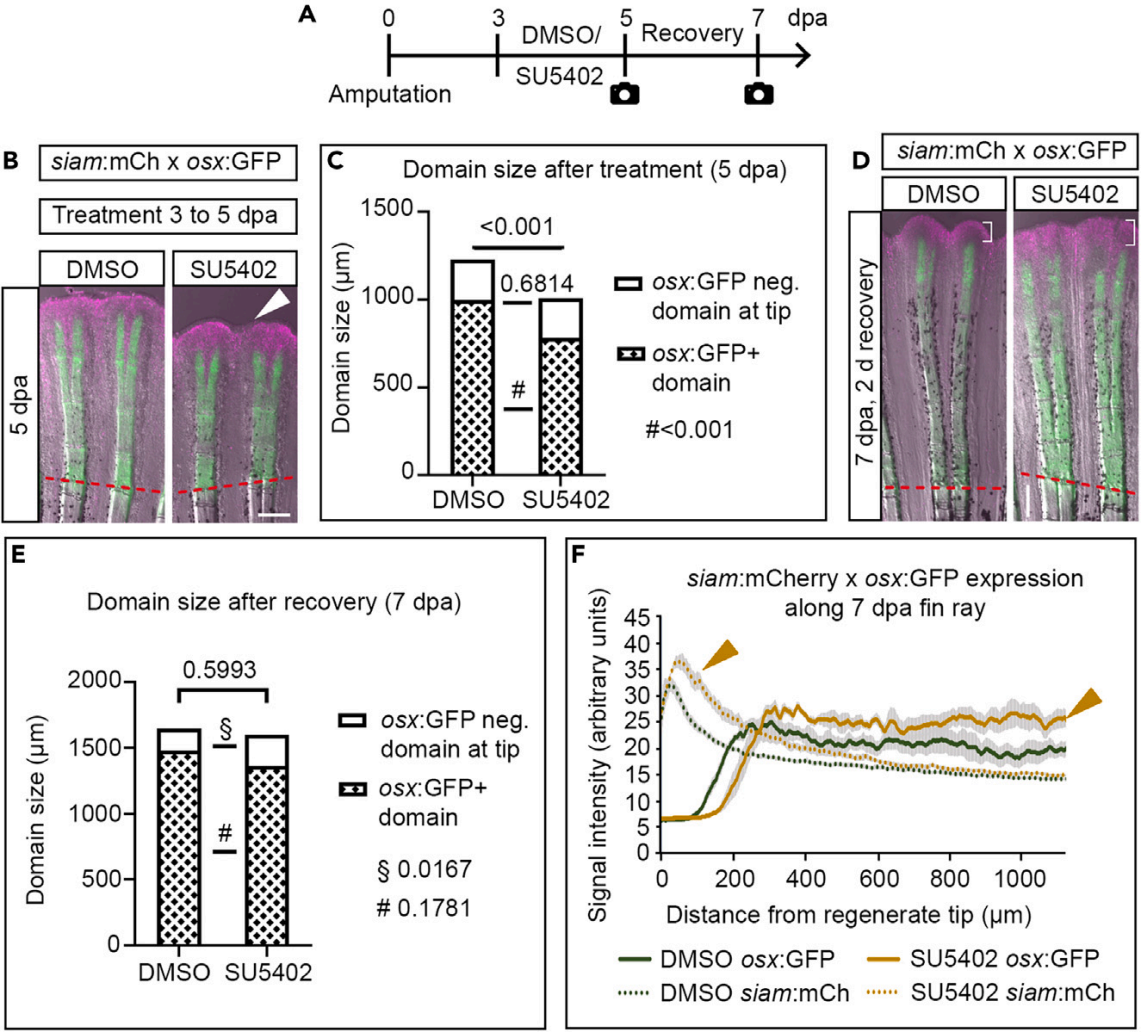
Effects of SU5402 on different regenerate domains (A) Treatment regimen. (B) 5 dpa fin regenerates of *siam*:mCherry x *osterix*:GFP transgenic zebrafish treated with SU5402 or DMSO from 3 to 5 dpa. White arrowhead, reduced regenerate length. (C) Quantification of experiment shown in (B). Welch’s t tests. (D) 7 dpa fin regenerates of *siam*:mCherry x *osterix*:GFP transgenic zebrafish treated with SU5402 or DMSO from 3 to 5 dpa and 2 days recovery (5–7 dpa). Brackets, *siam*+ tip region. (E) Quantification of experiment shown in (D). Welch’s t tests. (F) Fluorescence signal intensity of transgenic reporters along fin regenerate. Arrowheads, increased signal intensity. (B), (D) Scale bars 200 μm. (C), (E) Mean, (F) Mean ± SEM.

Next, we tested whether signaling pathway alterations would affect the size and function of these different regenerate domains (Wnt-active tip region versus *osterix* reporter+ domain). We turned to Fgf signaling, which is known to impact skeletal growth in various species^37^ and which is active in regenerating fins (Figures S4I and S9A).^26^^,^^38^^,^^39^^,^^40^^,^^41^ Suppression of Fgf signaling by SU5402 in *siam*:mCherry x *osterix*:GFP zebrafish from 3 to 5 dpa led to an overall reduced regenerate length (Figure 5A, arrowhead in Figure 5B). This reduction was attributable to a shortened *osterix*+ domain, while neither the size and Wnt reporter activity of the *osterix−*, *siam*+ tip region nor the activity of the *osterix*:GFP+ cells was affected (Figures 5A–5C and S9B). We let fins recover from the inhibitor treatment to test whether Fgf-suppressed regenerates would catch up in growth. Indeed, fins recovered regenerate length during a two-day recovery period in which both the size and the reporter activity of the distal Wnt-active domain increased (Figures 5D–5F). Furthermore, *osterix*:GFP signal intensity was higher in regenerates recovering from Fgf suppression, and the *osterix*:GFP domain had enlarged when compared to 5 dpa, suggesting enhanced osteoblast maturation (and potentially proliferation) during catch up (Figure 5F). These results indicate that the Wnt+ domain (encompassing DMB cells), pre-osteoblasts, and potentially committed osteoblasts compensate for impaired regenerate growth after Fgf signaling suppression which cannot erase the presence of positional identity in the regenerate.

### Osteoblast ablation impairs regeneration domain-specifically and regenerates can partially recover

**Figure 6:**
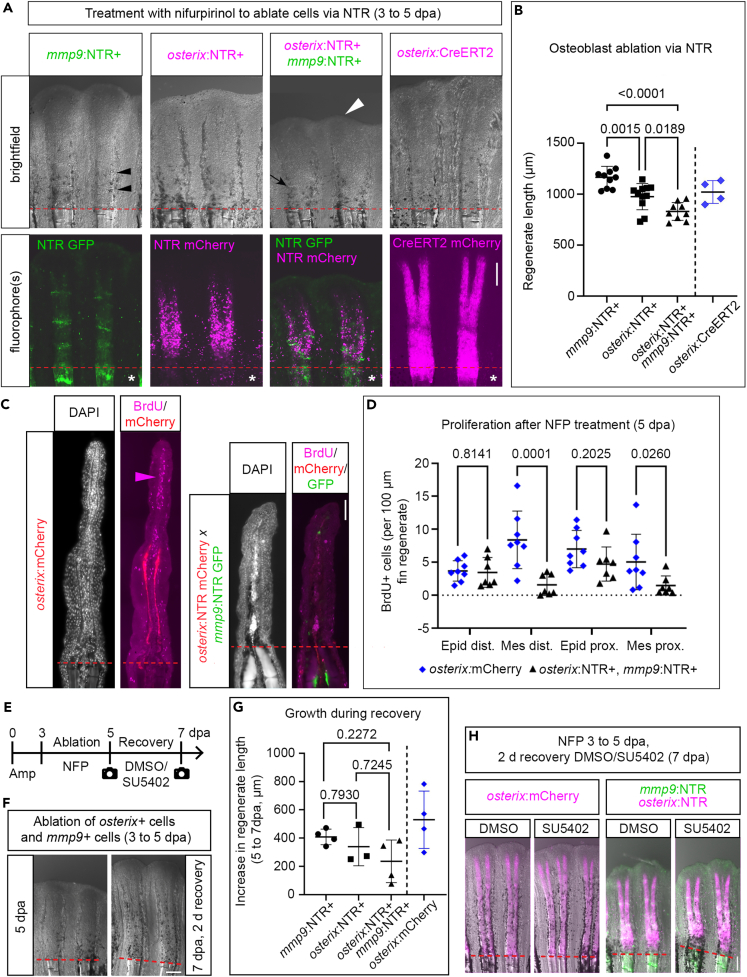
Effects of *mmp9*+ and *osterix*+ cell ablation on fin regeneration (A) 5 dpa fin regenerates of 3–5 dpa NFP-treated fin regenerates of *mmp9*:NTR, *osterix*:NTR, *osterix*:NTR x *mmp9*:NTR sibling zebrafish and *osterix*:CreERT2mCherry zebrafish, respectively. Fluorophore view images in high brightness and contrast settings (asterisks) to reveal dying cells in NTR+ zebrafish (same adjustments for *osterix*:CreERT2mCherry). White arrowhead, reduced regenerate length, black arrowheads, segment joints, black arrow, incomplete segment joint. (B) Quantification of experiment shown in (A). One-way Anova (Tukey), excluding *osterix*:CreERT2 (non-sibling). (C) IHC sections of NFP and BrdU treated 5 dpa *osterix*:mCherry and *osterix*:NTR x *mmp9*:NTR zebrafish fin regenerates. Arrowhead, BrdU+ cells in distal regenerate. (D) Quantification of experiment shown in (C). Kruskal-Wallis. Epid, epidermis, Mes, mesenchyme, dist., 0–250 μm from regenerate tip, prox., 250 μm from regenerate tip to amp plane. (E) Recovery treatment regimen. (F) 5 and 7 dpa fin regenerates of 3–5 dpa NFP treated fin regenerates of *osterix*:NTR x *mmp9*:NTR zebrafish (DMSO treatment during recovery). (G) Increase in regenerate length in *mmp9*:NTR, *osterix*:NTR, *osterix*:NTR x *mmp9*:NTR, and *osterix*:mCherry zebrafish during 2 days recovery (DMSO treatments). Dunnett’s T3, excluding *osterix*:mCherry (non-sibling). (H) DMSO and SU5402 recovery treated 7 dpa fin regenerates of *osterix*:NTR x *mmp9*:NTR and *osterix*:mCherry zebrafish. Scale bars (A, F, H) 200 μm, (C) 20 μm. (B), (D), (G) Mean ± SD.

We further investigated the contribution of different osteoblast populations to bone regeneration by ablating either *osterix*+ cells (proximal regenerate), *mmp9*+ cells (distal regenerate and joints), or both cell populations simultaneously. Transgenic zebrafish expressing nitroreductase (NTR) in the respective cells^7^^,^^8^^,^^42^ were treated with the substrate nifurpirinol (NFP),^43^ resulting in toxic product formation therein. *osterix*:NTR/*mmp9*:NTR double ablation from 3 to 5 dpa reduced regenerate length significantly when compared to individual *osterix*+ and *mmp9*+ cell ablation (Figures 6A and 6B). Individual *osterix*:NTR+ cell ablation had a stronger anti-regenerative effect than *mmp9*:NTR+ cell ablation. This confirmed the importance of *osterix*+ cells for bone regeneration and corroborated the finding that *osterix*+ and *mmp9*+ cells represent distinct fin regenerate cell populations.

We wondered how ablation affected proliferation in the regenerate and characterized the consequences of *osterix*+ and *mmp9*+ cell ablation by applying the S-phase marker Bromodeoxyuridine (BrdU) during the last 6 h of ablation. 5 dpa double-ablated fin regenerates showed a normal rate of proliferation in the epidermis but reduced mesenchyme (blastema and osteoblast) proliferation (Figures 6C and 6D), pointing to reduced cell expansion as one cause for impaired regeneration, in addition to death of ablated cells.

Next, we asked whether Fgf signaling would be pivotal in the recovery function of the distal regenerate after impairment of regeneration. To test this, we ablated *mmp9*+ and *osterix*+ cells from 3 to 5 dpa, determined regenerate length as well as *osterix*+ and *osterix−* domain sizes, and treated the zebrafish with SU5402 during the recovery phase thereafter (Figure 6E). An increase in regenerate length from 5 to 7 dpa was observed in control (DMSO) conditions without ablation and individual *mmp9*/*osterix* cell ablation, as well as after double ablation (Figures 6F, 6G, S10A, and S10B); however, addition of regenerative tissue in double-ablated fins lagged somewhat behind compared to single cell-type ablated fins (Figures 6G and S10B). Notably, Fgf inhibition did not affect recovery in any of the ablation conditions (Figures 6H, S10A, and S10B) indicating that Fgf signaling is dispensable for recovery function.

### *osterix*+ osteoblast ablation impairs bone matrix formation and patterning while *mmp9*+ cell ablation does not

**Figure 7:**
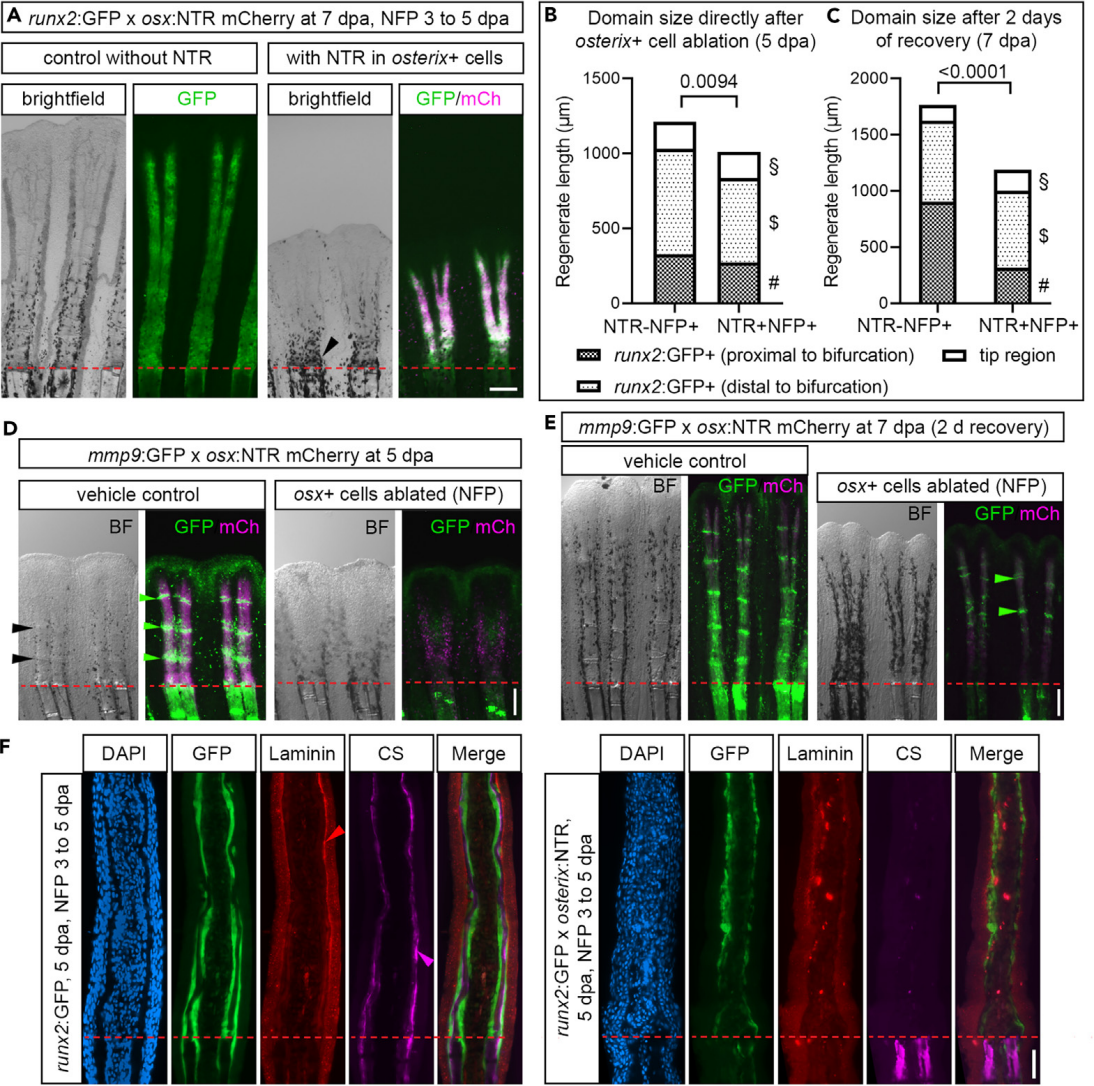
Structural integrity and establishment of segment joints are impaired after *osterix*+ cell ablation (A) 7 dpa fin regenerates of 3–5 dpa NFP treated *runx2*:GFP x *osterix*:NTR zebrafish. Arrowhead, accumulated melanocytes. (B) Regenerate length and domain sizes (proximal vs. distal to bifurcation, *runx2*:GFP negative tip region) at 5 dpa after NFP treatment. Welch’s t tests, Mann-Whitney test for tip region. p(§, tip region) = 0.7732, p($, dist. *runx2*:GFP) = 0.2564, p(#, prox. *runx2*:GFP) = 0.6341. (C) Regenerate length and domain sizes at 7 dpa, after 2days recovery. Welch’s t tests. p(§, tip region) = 0.0624, p($, dist. *runx2*:GFP) = 0.7599, p(#, prox. *runx2*:GFP) = 0.0100. (D) 5 dpa fin regenerates of 3–5 dpa DMSO and NFP treated *mmp9*:GFP x *osterix*:NTR zebrafish. Black and green arrowheads, segment joints. (E) 7 dpa fin regenerates of 3–5 dpa DMSO and NFP treated *mmp9*:GFP x *osterix*:NTR zebrafish (2 days recovery). Arrowheads, segment joints. (F) 5 dpa anti-chondroitin sulfate (CS) and anti-Laminin stained *runx2*:GFP x *osterix*:NTR fin regenerate sections after NFP treatment. Arrowheads, Laminin and CS signal in non-ablated fins. Red signal in ablated fins, dying *osterix+* cells. Scale bars (A, D, E) 200 μm, (F) 50 μm. (B), (C) Mean.

We went on to test whether cell ablation would irreversibly affect specific domains in the elongating regenerate. In order to distinguish recovering from non-recovering regions after ablation, we used the *runx2*:GFP+ reporter as an indicator for (pre-) osteoblast presence in *runx2*:GFP x *osterix*:NTR transgenic zebrafish at 5 dpa (i.e., after 2 days of NTR-mediated *osterix*+ cell ablation), and at 7 dpa after 2 days of recovery (Figures 7A–7C). At 5 dpa, *osterix*+ osteoblast-ablated fin regenerates were significantly shorter overall, while the length of individual domains (*runx2*:GFP+ region proximal to fin ray bifurcations, *runx2*:GFP+ region distal to fin ray bifurcations, tip region devoid of *runx2*:GFP signal) was only mildly reduced (Figure 7B). Two recovery days later, the distal *runx2*:GFP+ region showed normal domain length, while the *runx2*:GFP+ region proximal to bifurcation was significantly shorter and of the same length as that directly after ablation. In comparison, control-treated fins possessed a more than 2-fold longer pre-bifurcation proximal domain (Figures 7A and 7C). Furthermore, the size of the tip domain was slightly (albeit insignificantly) longer in fins that had undergone *osterix*+ osteoblast ablation. This indicates that distal regenerate regions less affected by *osterix*+ cell ablation regrow to pre-amputation size, independent of regeneration defects in proximal parts of the fin. It also suggests that distal regenerate cells might partly compensate for impaired regeneration of proximal fin tissue.

Next, we tested how *mmp9*:GFP+ cells, which partly reside at developing segment joints, would react to *osterix*+ cell ablation. In control treatment conditions, *mmp9*:GFP+ cells were visible in up to 3 forming segment joints as well as in the distal portion of the regenerate of *mmp9*:GFP x *osterix*:NTR reporter zebrafish (5 dpa, Figure 7D). In contrast, NFP-treated fin regenerates undergoing *osterix*+ cell ablation lacked confined expression of *mmp9*:GFP at segment joints and showed much weaker *mmp9* expression in a single broader domain at about 50% proximodistal level of the shorter fin regenerates, potentially reflecting dying cells (Figure 7D). At the same time, the segment joint indicator *pthlha* was not expressed in its usual pattern anymore (Figure S11A). Inspection of *osterix*+ cell ablated fins showed that segment joints did not form properly, although control-treated fins formed a minimum of one segment joint in a relatively proximal position (Figures 7D and S11B). Notably, in the reverse scenario of *mmp9*+ cell ablation, segment joint formation was observed (black arrowheads in Figures 6A and S11C), in agreement with a previous report on intact joints after *mmp9*+ cell ablation.^8^ This indicates pronounced patterning defects in case *osterix*+ osteoblasts are depleted from fin regenerates. We let *mmp9*:GFP x *osterix*:NTR regenerating zebrafish recover from vehicle/NFP treatment for 2 days. During this time, NFP-treated fins re-initiated segment joint formation in the distal part of the regenerate, resulting in an overall reduced segment joint number (Figures 7E and S11D), again indicating independence of the distal regenerate domain of proximal deterioration.

We asked why regenerate outgrowth and patterning were severely affected after *osterix*+ cell ablation and hypothesized that bone matrix secretion and maturation were diminished, leading to a lack of structural support in fin regenerates. Inspection of the sc RNA sequencing dataset suggested that chondroitin sulfate (CS), an indicator of matrix maturation,^44^ is produced by cells of the BLWE and non-osteoblast blastema and strongly in osteoblasts, as inferred from *chsy1* and *chpf2* expression (Figures S11E and S11F). Consequently, we stained for the presence of CS, in addition to investigation of the presence of Laminin as an indicator of basement membrane integrity of the BLWE overlying *osterix*+ osteoblasts.^45^^,^^46^ Ablation of *osterix*+ osteoblasts led to strong CS reduction in the regenerate while CS levels in stump bone matrix were unaffected (Figure 7F). Laminin expression was maintained after ablation; however, it showed irregular and ill-defined distribution indicating impaired BLWE integrity after ablation (Figure 7F). This indicates that structural ECM integrity is required for appropriate patterning of the different functional domains in the fin regenerate.

## Discussion

With this work, we provide a dataset on the transcriptomic landscape of rare mesenchymal (DMB, adjacent proximal blastema, osteoblast) and epidermal cell populations of the 3 dpa zebrafish fin regenerate browsable at the Single Cell Portal (https://singlecell.broadinstitute.org/) (access details, see STAR Methods) which represents a rich resource for researchers investigating appendage regeneration in teleost fish and other species.^47^^,^^48^^,^^49^ The dataset contains cells which may have been underrepresented in whole fin regenerate sequencing datasets due to the high prevalence of other cells^12^^,^^13^ but which are important to investigate the lineage specification of bone-forming cells and DMB cells serving as a signaling center throughout regeneration. Of note, the dataset does not contain mesenchymal cells located in the proximity of the amputation plane. As we have used cells from fin regenerates after repeated amputation, the dataset can also be used to study potential changes arising after recurrent injury, although our analysis suggests that gene expression in the distal regenerate is generally not altered between different rounds of amputation. This robustness is in line with previous reports investigating gene expression after repeated amputation.^50^^,^^51^

### Spatial arrangement and diversity of mesenchymal (DMB, adjacent proximal blastema, osteoblast) and BLWE cells in the regenerate

Research on the origin of skeletal cells in the regenerating appendage elucidated the role of stump tissues and cellular plasticity in the regeneration process.^4^^,^^5^^,^^6^^,^^8^^,^^9^^,^^47^ Other studies, such as by Brown et al.,^10^ Stewart et al.,^11^ and Wehner et al.^27^ have provided insight on the hierarchy and regulation of distal *Runx2*+ pre-osteoblasts, and a more proximal *osterix*+ osteoblast cell pool in the regenerate. Here, we uncovered the complex tissue constitution of the distal regenerate by identifying 5 osteoblast cell populations, including previously described committed osteoblasts, for which we present novel marker genes, two populations of proliferating osteoblasts, osteoblasts with a transcriptomic profile akin to (non-osteoblast) blastema cells, and joint cells located at the segment boundaries. We have analyzed a comparatively high number of osteoblasts (2,321 cells) of different maturity level and proliferative capacity and, as we suggest, proximodistal position. This proximodistal position (Figure 2J) can be inferred from combinatorial gene expression analysis. Importantly, we identified two populations of cells (Blastema3 and Osteo1), which form a transition zone between non-osteoblast blastema and osteoblast cell clusters of the regenerate. The observation that BLWE cell clusters separated completely from blastema and osteoblast cell clusters suggests that lateral BLWE do not contribute to the mesenchymal cell pool (and vice versa).

### Committed osteoblasts contribute to bone formation in the proximal regenerate and are supported by immature cells located at the tip

Here, we used genetic lineage tracing and ablation of *osterix*+ and *mmp9*+ cells, including Osteo4, Osteo1, and Osteo2 joint cells, to estimate the contribution of both cell populations to bone formation in the caudal fin regenerate. Thus, this work reveals their complementing contribution to regeneration and expands previous knowledge on the lineage restriction of *osterix*+ cells^4^^,^^7^ and the progenitor cell function of *mmp9*+ cells in the fin stump and regenerate.^8^ From 3 to 5 dpa, amplification of Osteo4 cells led to progeny covering ∼80% of the regenerate’s length demonstrating a considerable contribution, supported by a strong reduction in regenerate length after *osterix*+ cell ablation during regeneration. These observations are in line with an earlier report of impaired regeneration upon *osterix*+ cell ablation in the fin regenerate post-amputation (Figure S3C in^7^). At the same time, the restricted distal expansion of Osteo4 progeny suggested a somewhat limited renewal capacity, which is reflected by the partial expression of *pcna* in this population. In salamanders, transplantation experiments of different Cre-labeled populations (*Col1a2* vs. *Prrx1*) suggested that distal skeletal elements of the regenerated limb are derived from non-skeletal connective tissue cells.^47^ In our experiments, lineage tracing showed the absence of Osteo4 progeny from the distal 250 μm of the regenerate, suggesting alternative sources for this region. Candidate populations are *stmn1a*+ Osteo3 osteoblasts, which have a high proliferative activity (Figure S4G), and Osteo1 cells, which are found in the transition zone between blastemal and osteoblast cell clusters in our sc RNA sequencing dataset. Notably, strong *mmp9* expression is found in cells belonging to these and the Osteo0 clusters (Figure 3F), in addition to previously reported expression of *mmp9* in segment joint cells and epidermis.^8^ Occasional expansion of *mmp9*:CreERT2-converted cell clones at the very tip of the regenerate (Figures 3E and 3I) suggests contribution of *mmp9*+ cells to an osteoblast progenitor cell pool in this distal region of the regenerate but also hints at expansion of epidermal *mmp9+* cell populations.^8^ The hypothesis that, within the blastema, *mmp9*+ cells contribute to distal osteoblast progenitors is supported by the observed effects on simultaneous *mmp9*+ and *osterix*+ cell ablation, which decreased fin regenerate growth more dramatically than individual ablation. However, depletion of some *mmp9*-expressing myeloid cells in the fin regenerate^8^ might possibly add to the seen additive regeneration defects, as indicated by larval and adult macrophage ablation experiments during zebrafish tissue regeneration.^52^^,^^53^

Our results concerning the lacking expansion of *mmp9*+ cells at segment joints are unexpected in light of a prior study on the comprehensive potential of *mmp9*+ cells during fin regeneration. Ando et al.^8^ demonstrated a significant contribution of *mmp9*+ joint cells (of the fin stump) to newly forming fin regenerates. Fate mapping of the same cells in homeostatic fins resulted in broad labeling along the uninjured fin ray. A potential reason for this seeming discrepancy is the different experimental regimen that we have used by initiating a single recombination event post-amputation (not pre-amputation) with potentially much lower *mmp9* and *CreERT2* expression in joint-forming cells of the early regenerate. Furthermore, a different CreERT2 responder line (with however strong and ubiquitous reporter gene expression upon heat shock) was used. It is possible that *mmp9*+ joint-forming cells of the early regenerate are less potent than *mmp9*+ cells of the mature fin ray which might be indicated by the low fraction of proliferating Osteo2 cells at 3 dpa. Another seeming difference of our study and prior work concerns the consequences of NTR-mediated *mmp9*+ cell ablation.^8^ We noted a decreased regeneration potential (i.e., shorter fin regenerates) after *mmp9*+ plus *osterix*+ cell ablation within the early fin regenerate, while single *mmp9*+ cell ablation initiated *before* amputation did not lead to shorter fin regenerates in previous work.^8^ Again, experimental differences (ablation in the mature fin ray vs. the early regenerate) might cause the opposing observations. Notably, even though double ablation in the early regenerate has strong anti-regenerative effects, fins can recover once ablation treatment is discontinued. This supports the presence of other populations contributing to regeneration, and we suggest that distal blastema cells are of importance in this context.

The origin of osteoblast progenitors in the distal portion of the regenerate has been vague. Distal Runx2+ cells are maintained as a pre-osteoblast cell pool under the influence of Wnt signaling.^11^ In line with this, we identified a region of *runx2*:GFP+ pre-osteoblasts showing very weak GFP fluorescence in close proximity to the DMB. The respective cells often co-labeled with mCherry protein produced in *siam*:mCherry Wnt-responsive cells (Figure S8C). We suggest that these cells have just started to differentiate into pre-osteoblasts and that they are derived from (non-osteoblast) blastema cells. Alternatively, these cells may represent descendants of *runx2*:GFP+ osteoblasts that have lost *gfp* expression and have relocated distally. A second population of more proximal mCherry, GFP double+ cells may partly be explained by overlap of their respective transcript domains. However, no such zone of concomitant *gfp*/*mCherry* expression was detected in double transgenic *osterix*:GFP x *siam*:mCherry zebrafish, in agreement with low Wnt signaling in *osterix*+ osteoblasts.^11^^,^^27^ Nevertheless, a considerable amount of GFP/mCherry protein double+ cells were detected in the proximal region of the regenerate. We explain this discrepancy between mRNA and protein levels with a relocation of distal blastema cells to a more proximal position, or by a distal movement of osteoblasts toward the tip, resulting in mixing of cells. This is in line with our TEM data showing a gradual phenotype change of cells underlying the BLWE along the proximodistal direction. We conclude that a lineage dependence exists between (non-osteoblast) blastema cells and osteoblasts in the distal fin regenerate and that this transition is laid out by Blastema3 and Osteo1 cells, followed by proliferative Osteo0/3 cells. *mfap5*, *stmn1a*, *twist2*, and *runx2*:GFP are markers for this transition. Since blastema clusters were continuous in our analysis, actinotrichia-forming cells and even DMB cells may ultimately be involved in this process. Interestingly, photoconversion of DMB kaede-labeled cells, carried out by Wehner et al. (2014), led to a small population of converted cells localizing to a slightly proximal position (Figure S2I in^27^) and knockdown of the DMB “marker” *mmp13a* results in impairment of osteoblast commitment and differentiation in the regenerate.^29^ Altogether, we propose a model in which committed osteoblasts maintain themselves in the proximal domain of the regenerate, while the distal domain of the regenerate is populated by immature osteoblast progenitors derived from *mmp9*+ pre-osteoblasts, and *runx2*:GFP+ cells that are provided by distal blastema cells. Notably, this distal domain is able to compensate for diminished regenerate growth after a challenge such as Fgf signaling inhibition, demonstrating the adaptability of this region.

### The activity and size of the different regenerate domains depend on the presence of growth factors and reflect the need for continued regenerate growth

Here, we show that manipulation of growth factor signaling affects domain extension in the regenerate, similar to altered domain sizes after osteoblast ablation. Inhibition of Fgf signaling specifically reduced the domain size of *osterix*+ cells while leaving their reporter activity as well as the size of the *osterix−*, *siam*+ distal tip region unchanged during inhibition. This indicates a profound effect of Fgf signaling on newly differentiating *osterix*+ cells. This effect is reversible, and both the size and activity of the *siam*+ *osterix−* tip region and the activity of osteoblasts in the *osterix*:nGFP+ domain increase after discontinuation of Fgf suppression. Recovery of fin regenerate length is then completed within a few days, which is accompanied by the presence of an enlarged tip domain, suggesting intrinsic mechanisms detecting the need for enhanced regeneration which is likely carried out by enhanced proliferation at the junction of distal pre-osteoblasts and *osterix*+ osteoblasts. Notably, positional information cannot be overridden by Fgf suppression. The same is true for fins that have undergone a more dramatic, simultaneous ablation of both *mmp9*+ and *osterix*+ cells in fin regenerates. These fins catch up in distal growth when ablation is stopped, albeit at a somewhat slower pace than fins with single *mmp9*+ or *osterix*+ cell ablation (Figure 6G).

### Regenerate domains compensate for each other as long as structural support is provided

Here, we show that after ablation of *osterix*+ cells in the proximal regenerate, the *runx2*:GFP+ cell population distal to ray bifurcation expanded normally, and that the *runx2−* tip region remained slightly longer in *osterix*+ cell ablated fins than in control-treated fins. In contrast, the domain proximal to ray bifurcations, which did lose structural support, i.e., bone matrix, did not recover, therefore leading to a significantly reduced domain length. In the future, it will be important to test whether *osterix*+ cell ablated fins can grow to their pre-amputation size thanks to the tip region or whether regenerates remain significantly shorter because of the lack of proximal tissue. Given that the *runx2*:GFP− tip region is already slightly longer after a 2-day recovery, this region might take over “responsibility” for regenerate growth. This would be in agreement with other experimental settings we have tested, in which the tip region of the regenerate increased in size to make up for suppressed regenerate growth.

In our work, we detected patterning defects affecting segment joint formation in case *osterix*+ osteoblasts lining segments (but not segment boundaries) were ablated. We did not see segment joint formation defects in case of *mmp9*+ cell (i.e., joint forming cell) ablation, as reported previously.^8^ This suggests that *osterix*+ cells and the resulting deposited bone matrix are required for appropriate segmentation of the fin. Altogether, this demonstrates a pivotal function of committed osteoblasts, but also pre-osteoblasts and distal blastema cells, for growth, bone formation, and patterning of the regenerating vertebrate appendage.

### Limitations of the study

Here, we have analyzed selected fin regenerate cell populations via FACS enrichment and sc RNA sequencing. A detailed investigation of other fin regenerate mesenchyme populations (i.e., fibroblasts and osteoblasts found close to the amputation plane) along with distal WE cell populations is lacking. Furthermore, genetic fate mapping of DMB cells via CreERT2 should be performed, along with testing for potential dissimilarities of available Cre responder lines. In the future, tissue-specific inhibition of Fgf signaling during regeneration is desirable.

## STAR★Methods

### Key resources table

**Table:** 

REAGENT or RESOURCE	SOURCE	IDENTIFIER
**Antibodies**		

Chicken anti-GFP	Abcam	#catab13970; RRID:AB_300798
Mouse anti-mCherry	Takara Bio	#cat632543; RRID:AB_2307319
Rabbit anti-dsRed	Takara Bio	#cat632496; RRID:AB_10013483
Mouse anti-chondroitin sulfate	Sigma-Aldrich	#catC8035; RRID:AB_476879
Rabbit anti-Laminin	Sigma-Aldrich	#catL9393; RRID:AB_477163
Rat anti-BrdU	Novus-Bio	#catNB500-169; RRID:AB_10002608
Goat anti-chicken-Alexa 488	Thermo-Fischer	#catA-11039; RRID:AB_2534096
Goat anti-mouse-Alexa 555	Thermo-Fischer	#cat A-21424; RRID:AB_141780
Goat anti-rabbit-Alexa 555	Thermo-Fischer	#cat A-21428; RRID:AB_2535849
Goat anti-mouse-Alexa 633	Thermo-Fischer	#cat A-21046; RRID:AB_2535715
Goat anti-rat-Alexa 633	Thermo-Fischer	#cat A-21094; RRID:AB_2535749
Goat anti-rabbit-Alexa 633	Thermo-Fischer	#cat A-21071; RRID:AB_2535732
Anti-Digoxigenin-AP, Fab fragments	Roche	#cat 11093274910; RRID:AB_2313640

**Biological samples**		

Zebrafish fin regenerates	See Experimental models	See Table S9

**Chemicals, peptides, and recombinant proteins**		

4-hydroxytamoxifen	Sigma	#catH7904
Nifurpirinol	Sigma	#cat#32439
SU5402 (H3B-6527)	Selleckchem	#catS8675
5-Bromo-2‘-deoxyuridine (BrdU)	Sigma-Aldrich	#catB5002
Collagenase-dispase	Roche	#cat10269638001
Calcein violet	Invitrogen	#catC34858
HBSS solution (without CaCl_2_ and MgCl_2_)	Gibco	#cat12082739
SIGMAFAST™ Fast Red TR/Napthol AS-MX Tablets	Sigma	#cat F4648
NBT/BCIP Stock Solution	Roche	#cat 11681451001
T7 RNA Polymerase	Roche	#cat10881767001
SP6 RNA Polymerase	Roche	#cat10810274001
T3 RNA Polymerase	Roche	#cat11031163001
Fluorescein RNA Labeling Mix	Roche	#cat32874620
Digoxigenin RNA Labeling Mix	Roche	#cat53119620
Single cell 3’ RNA-seq v2	Chromium, 10X Genomics	https://assets.ctfassets.net/an68im79xiti/RT8DYoZzhDJRBMrJCmVxl/6a0ed8015d89bf9602128a4c9f8962c8/CG00052_SingleCell3_ReagentKitv2UserGuide_RevF.pdf

**Critical commercial assays**		

Zero Blunt TOPO PCR Cloning Kit	Invitrogen	#cat450245

**Deposited data**		

scRNAseq dataset	Gene expression ombnibus (GEO)	GEO: https://www.ncbi.nlm.nih.gov/geo/query/acc.cgi?acc=GSE251828
scRNAseq dataset	Single Cell Portal	SCP: https://singlecell.broadinstitute.org/single_cell/study/SCP1674
Source code for scRNA expression analysis	Zenodo	Zenodo: https://doi.org/10.5281/zenodo.10245884

**Experimental models: Organisms/strains**		

*siam:mCherry = Tg(7xTCF-Xla.Sia:NLS-mCherry)*^*ia5*^	Moro et al.^54^	https://doi.org/10.1016/j.ydbio.2012.03.023
*shh:GFP = Tg(-2.7shha:GFP)*^*t10*^	Neumann et al.^55^	https://doi.org/10.1126/science.289.5487.2137
*osterix:CreERT2-p2a-mCherry=Tg(Ola.Sp7:CreERT2-p2a-mCherry)*^*tud8*^	Knopf et al.^4^	https://doi.org/10.1016/j.devcel.2011.04.014
*runx2:GFP=Tg(Hsa.RUNX2-Mmu.Fos:EGFP)*^*zf259*^	Knopf et al.^4^	https://doi.org/10.1016/j.devcel.2011.04.014
*osterix:mCherry=Tg(Ola.Sp7:mCherry)*^*zf131*^	Spoorendonk et al.^56^	https://doi.org/10.1242/dev.024034
*osterix:GFP=Tg(Ola.Sp7:NLS-GFP)*^*zf132*^	Spoorendonk et al.^56^	https://doi.org/10.1242/dev.024034
*mmp9:GFP=TgBAC(mmp9:EGFP)*^*tyt206*^	Ando et al.^8^	https://doi.org/10.1016/j.devcel.2017.10.015
*mmp9:NTR=TgBAC(mmp9:EGFP-NTR)*^*tyt207*^	Ando et al.^8^	https://doi.org/10.1016/j.devcel.2017.10.015
*mmp9:CreERT2=TgBAC(mmp9:Cre-ERT2, cryaa:EGFP)*^*tyt208*^	Ando et al.^8^	https://doi.org/10.1016/j.devcel.2017.10.015
*dsRed2GFP=Tg(hsp70l:loxP-DsRed2-loxP-nlsEGFP)*^*tud9*^	Knopf et al.^4^	https://doi.org/10.1016/j.devcel.2011.04.014
*Actb:DsRed2GFP=Tg(Ola.Actb:LOXP-DsRed2LOXP-EGFP)*^*tyt201Tg*^	Yoshinari et al.^36^	https://doi.org/10.1111/dgd.12013
*osterix:NTR=Tg(Ola.Sp7:mCherry-Eco.NfsB)*^*pd46*^	Singh et al.^7^	https://doi.org/10.1016/j.devcel.2012.03.006

**Oligonucleotides**		

Probe design	This paper	See Table Oligonucleotides
*Lum*	This paper	N/A
*sgk1*	This paper	N/A
*stmn1a*	This paper	N/A
*sgms2*	This paper	N/A
*fgfbp2a*	This paper	N/A
*ifitm5*	This paper	N/A
*panx3*	This paper	N/A
*col17a1b*	This paper	N/A
*mfap5*	This paper	N/A
*mustn1a*	This paper	N/A
*mmp13a*	This paper	N/A
*postna*	This paper	N/A
*mxra8b*	This paper	N/A
*abi3bpb*	This paper	N/A
*fgl1*	This paper	N/A
*spon1b*	This paper	N/A
*twist2*	This paper	N/A
*ednrab*	This paper	N/A
*timp2b*	This paper	N/A
*zic2a*	This paper	N/A
*kpna2*	This paper	N/A
*LOX (1 of many)*	This paper	N/A
*gstm.3*	This paper	N/A

**Software and algorithms**		

Cellranger 3.0.0	10X Genomics	https://support.10xgenomics.com/single-cell-gene-expression/software/pipelines/latest/using/count)
Scanpy1.6.0	scanpy	https://github.com/scverse/scanpy/releases/tag/1.6.0
Anndata2ri	Theis Lab, anndata2ri	https://github.com/theislab/anndata2ri
Scvelo	Theis Lab	https://github.com/theislab/scvelo
edgeR	Bioconductor	https://bioconductor.org/packages/release/bioc/html/edgeR.html
Limma	Bioconductor	https://bioconductor.org/packages/release/bioc/html/limma.html
Custom software stack and source code for single cell analysis	This paper	https://doi.org/10.5281/zenodo.10245884
Illustrator	Adobe	https://www.adobe.com/de/products/illustrator.html
Photoshop	Adobe	https://www.adobe.com/de/products/photoshop.html
Graphpad Prism 6.0	Graphpad Software	https://graphpad-prism.software.informer.com/6.0/
Affinity Photo	Affinity	https://affinity.serif.com/de/
Affinity Designer	Affinity	https://affinity.serif.com/de/
ZEN 2012 (blue edition)	Zeiss	https://zen-2012-blue-edition.software.informer.com/
ImageJ	Fiji	https://imagej.net/software/fiji/downloads

### Resource availability

#### Lead contact

Requests for further information, resources and reagents should be directed to the lead contact, Franziska Knopf [franziska.knopf@tu-dresden.de, phone +49 (0) 351 458-82303].

#### Materials availability

Reagents generated in this study will be shared by the lead contact upon request.

#### Data and code availability

• The single cell RNA sequencing dataset is publicly available at the Single Cell Portal and GEO. The accession numbers are listed in the key resources table.
• The source code for the single cell gene expression analysis is publicly available on Zenodo and the DOI is listed in the key resources table.
• Additional information regarding the data reported in this paper is available from the lead contact upon request.

### Experimental model and a participant details

#### Animal experiments

All procedures were approved by and performed according to the animal handling and research regulations of the Landesdirektion Dresden (Permit numbers: AZ 24D-9168.11-1/2008-1, AZ 24-9168.11-1/2011-52, AZ DD24.1- 5131/354/87, AZ DD24.1-5131/450/4, AZ 25-5131/496/56 and respective amendments).

#### Transgenic zebrafish and husbandry

The transgenic fish lines used in this study have been described: *siam*:mCherry=Tg(*7xTCF-Xla.Sia*:NLS-mCherry)^ia5 54^, *shh*:GFP=Tg(*-2.7shha*:GFP)^t10 55^, *osterix:*CreERT2-p2a-mCherry*=*Tg(*Ola.Sp7*:CreERT2-p2a-mCherry)^tud8^, *runx2*:GFP=Tg(*Hsa.RUNX2-Mmu.Fos*:EGFP)^zf259 4^, *osterix*:mCherry=Tg(*Ola.Sp7*:mCherry)^zf131^, *osterix*:GFP=Tg(*Ola.Sp7*:NLS-GFP)^zf132 56^, *mmp9*:GFP=TgBAC(*mmp9*:EGFP)^tyt206^, *mmp9*:NTR=TgBAC(*mmp9*:EGFP-NTR)^tyt207^, *mmp9*:CreERT2=TgBAC(*mmp9*:Cre-ERT2, cryaa:EGFP)^tyt208 8^, dsRed2GFP=Tg(*hsp70l*:loxP-DsRed2-loxP-nlsEGFP)^tud9 4^, *Actb*:DsRed2GFP=Tg(*Ola.Actb*:LOXP-DsRed2LOXP-EGFP)tyt201Tg,^36^ dsRed2GFP∗=Tg(*hsp70l*:loxP-DsRed2-loxP-nlsEGFP)^tud9 4^ x Tg(*Ola.Actb*:LOXP-DsRed2LOXP-EGFP),^36^ *osterix*:NTR=Tg(*Ola.Sp7*:mCherry-Eco.NfsB)^pd46 7^. The artificial promoter *siam* drives mCherry expression in Wnt responsive cells in the DMB and some more proximal cells.^54^ The *shha* promoter element^55^ activates expression of GFP in BLWE cells. Osteoblasts of different maturity were labeled by the activity of *osterix* and *RUNX2* promoter elements.^4^ The dsRed2GFP line drives ubiquitous expression of dsRed2 and reliable expression of nGFP after CreERT2-mediated sequence recombination and heat shock.^4^ Fish were bred and maintained as described.^4^ All transgenic zebrafish lines used in this study are listed in the key resources table. Adult fish of both sexes were used for all experiments.

### Method details

#### Fin clips

Fish were anesthetized in Tricaine 0.02% (Sigma-Aldrich, #A5040) and the caudal fin was resected at 50 % of its length using a scalpel. Animals were transferred to fish water and allowed to regenerate at 28°C.^41^ 3, 5 and 7 dpa regenerates were obtained from fin-clipped male and female zebrafish.

#### Tissue dissociation and flow cytometry

Fin regenerates of quadruple transgenic fish (*siam:*mCherry*, shh:*GFP*, osterix:*CreERT2-p2a-mCherry*, runx2:*GFP*)* were harvested at 3 dpa, cut into small pieces with a scalpel and transferred into 1 ml collagenase-dispase solution (1 mg/ml in PBS, Roche #10269638001) for 10 min at 28°C. The sample was pipetted slowly up and down with an elongated, flame polished Pasteur pipette. The procedure was repeated 4 times with decreasing inner tip diameters of the Pasteur pipettes until a homogenous solution was obtained. The dissociates were poured onto an equilibrated 70 μm cell strainer and collected in 10 ml HBSS solution (without CaCl_2_ and MgCl_2_, Gibco #12082739). After centrifugation (15 minutes, 1800 rpm, 4°C), the supernatant was discarded and the remaining cell pellet resuspended in 500 μl 2 % BSA in PBS. Calcein violet (1μl 10mM, Invitrogen #C34858) was added to the cell solution and incubated for 30 minutes. Calcein violet, GFP and mCherry+ cells were collected in 50 μl 2% BSA in PBS via fluorescence-activated cell sorting (BD LSR Fortessa) and processed for single-cell RNA sequencing analysis based on 10X Genomics (10X Chromium system, 10X library preparation according to the manufacturer's instructions).

#### Single-cell RNA sequencing analysis

For each experiment about 8000 cells from zebrafish fin regenerates were flow-sorted into BSA-coated PCR tubes containing 1 μl of PBS with 0.04 % BSA. All cells were carefully mixed with reverse transcription mix before loading them in a Chromium Single Cell A Chip on the 10X Genomics Chromium controller^57^ and processed further following the guidelines of the 10X Genomics user manual for single cell 3’ RNA-seq v2. In short, the droplets were directly subjected to reverse transcription, the emulsion was broken and cDNA was purified using silane beads. After amplification of cDNA with 12 cycles, it underwent a purification with 0.6 volume of SPRI select beads. After quality check and quantification using the Fragment Analyzer (Agilent), 30 ng cDNA were used to prepare sc RNA-seq libraries - involving fragmentation, dA-Tailing, adapter ligation and a 12 cycles indexing PCR based on manufacturer’s guidelines. After quantification, both libraries were sequenced on an Illumina Nextseq500 system in paired-end mode with 26 bp/57 bp (for read 1 and 2 respectively), thus generating ∼60-90 mio. fragments for the transcriptome library on average.

A custom reference based on GRCz10, Ensembl annotation e98 was created, by first adding the sequences of *gfp* and *mCherry* as separate chromosomes to the fa file and the gtf file and then building the cellranger reference using cellranger mkref. Next, fastq files were processed with cellranger count from 10X genomics (https://support.10xgenomics.com/single-cell-gene-expression/software/pipelines/latest/using/count) version 3.0.0 using the custom reference. This resulted in a dataset with 2532, 3601, 2481 and 3413 mean counts per cell and median number of 300, 532, 975 and 508 detected genes per cell, and 7028, 4707, 2846 and 4535 cells for regeneration experiments Reg1, Reg2, Reg3 and Reg4, respectively.

For the downstream analysis of the 10X data, current best practices were followed.^14^ The filtered gene counts matrices were read with scanpy 1.6.0.^58^ Next, cells were filtered based on the number of total counts, the number of detected genes and with sample specific thresholds: Only cells with more than 2000, 1000, 1000 and 1000 total counts as well as more than 600, 300, 300 and 400 detected genes for samples Reg1, Reg2, Reg3 and Reg4, respectively, were kept for the analysis. Furthermore, cells with more than 5% of mitochondrial reads were filtered. After quality control, our dataset consisted of 1342, 1410, 2102 and 1814 cells from the respective regeneration experiment. Only genes which were detected in more than 3 cells (counting all samples together) were kept for downstream analysis. Normalization was performed with the scanpy function sc.pp.normalize_total and the data was log-transformed with sc.pp.log1p. Highly variable genes were detected with sc.pp.highly_variable_genes setting n_top_genes=4000. Principal component analysis was performed on the highly variable genes. A neighbor graph was constructed with sc.pp.neighbors setting n_neighbors=30 and n_pcs=15. Next, a UMAP was constructed with sc.tl.umap setting min_dist=0.9.^59^ Clustering was done using sc.tl.leiden with resolution=0.2. Marker genes were computed with sc.tl.rank_genes_groups. Two clusters were identified as blood vessels and immune cells (autofluorescent cells) and those were excluded from further analysis, leaving 1219 (Reg1), 1331 (Reg2), 2007 (Reg3) and 1705 cells (Reg4). Next, principal component analysis, neighborhood graph construction, UMAP computation and marker gene detection were repeated with the same functions and parameters as above. In result, the presented UMAPs show 691 BLWE cells (Basal), 3250 (non-osteoblast) blastema cells (Blastema), and 2321 osteoblasts (Osteo). The 3 main clusters were sub-clustered using sc.tl.leiden setting the restrict_to parameter to the respective cluster. The resolution parameter was set to 0.25, 0.1 and 0.35 for the blastema, BLWE and osteoblast clusters, respectively. For each sub-cluster, marker genes were computed as above but using only the cells of the respective main cluster as a reference. Trajectory inference was performed with sc.tl.paga and the paga plot was created sc.pl.paga_compare setting threshold=0.1.^15^ To compute RNA velocities, we used the velocyto.py command line interface on the cellranger bam files to create a loom file that contains spliced and unspliced reads.^60^ We read this loom file with scvelo and merged it with the AnnData object that was created by the scanpy analysis above.^61^ The resulting data was processed according to the scvelo tutorial: In detail, filter_and_normalize was run with min_shared_counts=20 and n_top_genes=2000, next moments were computed, and the velocities were computed with mode “stochastic”. A velocity graph was computed and the result was visualised on the UMAP using pl.velocity_embedding_stream. For differential expression analysis, we used a limma-voom workflow: only highly expressed genes were considered, i.e. genes with a cpm value>1 in more than 25% of the cells.^62^ The AnnData object was converted to an edgeR DGEList object using anndata2ri (https://github.com/theislab/anndata2ri) and scran.^63^^,^^64^ Normalisation factors of the raw count matrix were computed with edgeR’s calcNormFactors function. The number of detected genes per cell was scaled to zero mean and unit variance and added as a co-factor to the design formula. The other factor that was added was the condition (Reg1 vs Reg2-4 pooled together). Next, the DGE list was transformed using limma’s voom function.^65^ Next, edgeR’s functions lmFit, contrasts.fit, treat (with lfc=log2(1.5)) and topTreat were used to generate a list of differentially expressed genes. Next, pseudospace coordinates were computed. For the lateral coordinate, the gene set *lamb1a*, *wnt5b*, *shha* and *phlda2* were used. A laterality score was computed using the scanpy function sc.tl.score_genes. Next, the transcriptome data object was subset to the lateral gene set. Diffusion pseudotime was computed by running sc.pp.neighbors with n_neighbors=50, sc.tl.diffmap and sc.tl.dpt on the subsetted transcriptome data. For sc.tl.dpt, the cell with the highest laterality score was used as the root. The lateral coordinate of pseudospace was then computed by a rank transformation of the pseudotime. For the distal coordinate, the same computation was performed using the gene set *aldh1a2*, *wnt5a*, *fgf3*, *fgf10a*, *igf2b*, *msx3*, *msx1b*, *cdh4*, *dkk1b*, *wnt3a*, *dkk1a*, *dlx5a*, *junba*, *junbb*, *msx2b*, *spry4* and *wnt10a*.

#### RNA *in situ* hybridization and histology

The plasmids to obtain probes for *fgf24* (restriction digest with NotI, transcription with T7), and *pthlha* (restriction digest with BamHI, transcription with SP6) have been published.^32^^,^^66^ *mCherry* (restriction digest with HindIII and transcription with T7), and *gfp* (restriction digest with EcoRI, transcription with T3) plasmids were provided by Gilbert Weidinger. The *CreERT2* probe plasmid (restriction digest with AgeI, transcription with T7) was provided by Stefan Hans. The *tnc in situ* probe plasmid was generated by René Bernitz and provided by Daniel Wehner. The *pthlha in situ* probe plasmid^32^ was provided by Marie-Andrée Akimenko. Additional probes have been synthesized from pCR-Blunt II-TOPO vectors after cloning of the respective sequences by using the Zero Blunt TOPO PCR cloning Kit (Invitrogen) according to the manufacturer's instructions. The forward and reverse oligonucleotides to amplify the cDNA sequences are listed below (**Table Oligonucleotides**), along with the corresponding fragment sizes.

**Table:** Table Oligonucleotides. Primers used to amplify cDNA sequences

gene	forward oligo (5->3)	reverse oligo (5->3)	fragment size [bp]
*lum*	CTACATACCCTCCGCACCAC	CGGGATGGTCTTCAGCTTGT	751
*sgk1*	ACCTGACACCACCACAAGATG	TGCACAGGCCAAAGTCAGTC	558
*stmn1a*	CGAACTTGACTTGCATTGAGGT	CTCAGTCTTGTACAAAGAACAGTCA	700
*sgms2*	TGCCATCGGAATGGTGGAAG	GAGGTACGTGAGGGTGAGGA	500
*fgfbp2a*	CAGAAACCCATGCCAAAGCC	TAGCAGGGAGTGATTTCGCC	787
*ifitm5*	TCTTCCAGGAGTTTGGACCG	ACATGGACATGAATTAGGGAACAAA	604
*panx3*	AGTGAAGCAGGCAGCCTATG	GGAGCAGATGGCCCTTAGAC	835
*col17a1b*	CTGGAGTCCTAACGTCCAGC	GTGTGGGCATTCATGGAGGA	787
*mfap5*	TGAAGACCCTGAAAGATGGGC	CACAGAGACATTCTGACGAGCTT	584
*mustn1a*	ACCAGCAGCACAAACCAAAAAC	ACGTGTTTCAGCAGTATGATCC	541
*mmp13a*	CGGTGCACTCATGTATCCCA	CCCAAAAACCAGCGTTCAGAC	934
*postna*	TGACAGCGTTTATGGCACCT	GCGGCAGAAAGACCAAGTTC	894
*mxra8b*	CAGTAGGGTCGGATGTGGTT	TGATGGTGAAGATGGCAGGAA	703
*abi3bpb*	CTGTTCAGCCAATGCCAACC	TGCTGAAGAACCCCCATGTC	947
*fgl1*	TGCTTTGCACTCTGGATTTG	ATCCATTCAGGTTGGCAGAG	765
*spon1b*	TCCGAGTATGGATCACCCGT	TGGGAACTGCTTGACGTACC	822
*twist2*	GCAGAAAAGGTTCGGGAGGA	TCCATGCAGCGTACTTCAAGA	872
*ednrab*	GTGGCCGTTTGATGACAGTG	CGACCGGAAGCAGTTCTTGA	712
*timp2b*	TTGGTCGTGAAGAGTGTCCG	TGGTAGCTGCGTTTGGGATT	726
*zic2a*	CGAGATGCTTCAAGAACCAGGA	TGAGAATGATGGAGCGAGCC	736
*kpna2*	AATGTGTGACCCACGTCTTCA	CCAGCGATGTTTCCAAGAGC	754
*LOX (1 of many)*	AGGTTTCTGTTCTCCTGTTCCC	ACGTGTCGTAACAACCAGGA	994
*gstm.3*	ATTCATCTCCGCTCACTGCT	AGGTGAACCTACAGCTGAAAAG	683

For whole mount RNA *in situ* hybridization, fin regenerates were fixed in 4 % PFA in PBS overnight at 4°C. After fixation, fins were washed in PBS and dehydrated in methanol for storage at -20°C. Fins were rehydrated gradually in methanol solutions in PBT (PBS, 0.1% Tween 20) of 75 %, 50 % and 25 % at room temperature for 5 minutes each and washed with PBT (4x 5 minutes). Fins were digested for 20 minutes using Proteinase K (Roche, 03115879001) (20 μg/ml). Fins were rinsed in PBT, refixed for 15 minutes in 4 % PFA in PBS and washed with PBT (5x 5 minutes) at room temperature. Samples were incubated in hybridization buffer [5x SSC final concentration from 20x SSC stock (0.3 M sodium citrate, 3 M sodium chloride, pH 7), 500 μg/ml torula yeast RNA (Sigma), 50 μg/ml heparin, 0.1 % Tween 20, 9 mM citric acid pH 6.0, 50 % deionized formamide] for 2 h at 65°C in a water bath before hybridization with the probes overnight at 67°C. On the next day, the samples were washed at 67°C with hybridization buffer (20 minutes), 1:1 SSCT 2x (2x SSC, 0.1% Tween 20)/deionized formamide (3x 20 minutes), SSCT 2x (2x for 20 minutes) and in SSCT 0.2x (3x 30 minutes). After a 5-minute wash in PBT, the samples were incubated at room temperature in blocking buffer [5% sheep serum (Sigma, S2263) +10 mg/ml BSA (bovine serum albumin), in PBT] for 1 h before incubating them overnight in blocking buffer with Anti-Digoxigenin-AP antibody (Roche, 11093274910, 1:4000) at 4°C. The Anti-Digoxigenin-AP antibody was removed, the samples were washed with PBT (6x 20 minutes) and NTMT (100 mM Tris HCl pH 9.5, 50 mM MgCl_2_, 100 mM NaCl, 0.1 % Tween 20) (3x 5 minutes). The samples were stained using NBT/BCIP (Roche, 11681451001) staining solution (20 μl NBT/BCIP per ml NTMT) in the dark until signal was visible. The staining reaction was stopped by rinsing two times in PBT and adding STOP solution (0.05M phosphate buffer pH 5.8, 1mM EDTA, 0.1% Tween 20). Afterwards, samples were cleared using 100 % ethanol (2x 15 minutes), 50 % ethanol (5 minutes), washed in PBT (4x 5 minutes) and transferred into 80 % glycerol / 20 % STOP solution for imaging.

For double RNA ISH, NBT/BCIP staining (stock solution Roche, 11681451001) was combined with Fast Red staining for simultaneous detection of two transcripts. After staining with NBT/BCIP, fins were briefly rinsed twice in PBT and transferred to new tubes. This was followed by incubation in 0.1M Glycin/HCL, pH 2,2 + 0.1% Tween (2x 5 minutes) and washing with PBT (4x 5 minutes). 200μl Anti-Fluorescein-AP, Fab fragments antibody (Roche, 11426339810) was added (1:2000 dilution in PBT + 2 mg/ml BSA + 2% sheep serum) and incubated over night at 4°C. The fins were kept dark and washed in PBT (2x 5 minutes, 6x 30 minutes). Fins were then washed in 0.1M Tris pH 8.2 + 0.1% Tween (3x 5 minutes). SIGMAFAST™ Fast Red TR/Napthol AS-MX Tablets (Sigma, F4648) were dissolved according to the manufacturer's instructions. Specimens were kept dark and at room temperature in 500 μl Fast Red staining solution until the staining had developed. The staining reaction was stopped by two brief washes with PBT and one wash in STOP solution. Fins were stored in 80% glycerol/20% STOP solution thereafter and processed for cryosectioning. Cryosections were imaged with a Zeiss 10x/0.45 Plan-Apochromat air objective on an ApoTome1 equipped with a Zeiss AxioCam MRc color CCD camera and a Zeiss ZEN blue (v 2012) software.

#### Immunohistochemistry

Preparation of tissue for cryosectioning and immunofluorescence were performed as described.^4^ 12 μm cryosections were obtained with a Cryostat HM560. Primary antibodies used were: chicken anti-GFP (Abcam, ab13970) at 1:2000, mouse anti-mCherry (Clontech, 632543) at 1:450, rabbit anti-dsRed (Clontech, 632496) at 1:300, mouse anti-chondroitin sulfate (Sigma, C8035) at 1:300, rabbit anti-Laminin (Sigma, L9393) at 1:200, rat anti-BrdU (Novus Bio, NB500-169) at 1:300. Secondary antibodies used were: goat anti-chicken-Alexa 488 (Thermo Fisher, A-11039), goat anti-mouse-Alexa 555 (Thermo Fisher, A-21424), goat anti-rabbit-Alexa 555 (Thermo Fisher, A-21428), goat anti-mouse-Alexa 633 (Thermo Fisher, A-21046), goat anti-rat-Alexa 633 (Thermo Fisher, A-21094) at 1:1000.

#### Combined RNA *in situ* hybridization and immunohistochemistry

RNA *in situ* hybridization was performed as indicated previously but after incubation of anti-DIG-AP, primary antibodies (chicken anti-GFP, Abcam, ab13970, 1:2000 and rabbit anti-DsRed, Clontech, 632496, 1:200) were incubated overnight at 4°C. Fins were washed in PBT and incubated with secondary antibodies (goat anti-chicken-Alexa 488, Thermo Fisher, A-11039 and goat anti-rabbit-Alexa 633, Thermo Fisher, A-21071) at 1:500. After immunohistochemistry, fins were stained with Fast Red solution and cryosectioned. Cryosections were imaged with a Zeiss 20x/0.8 Plan-Apochromat air objective on a LSM 980/MP inverse equipped with a Zeiss Zen2 blue (v 3.0.79.00004 HF4) software.

#### Fate mapping

Transgenic fish (*osterix*:CreERT2-p2a-mCherry x *hsp70l*:R2nlsGFP; *mmp9*:CreERT2, *cryaa*:EGFP x *hsp70l*:R2nlsGFP and *osterix*:CreERT2-p2a-mCherry x *mmp9*:CreERT2, *cryaa:EGFP* x *hsp70l*:R2nlsGFP x *Actb*:DsRed2GFP)^4^^,^^8^^,^^36^ were injected intraperitoneally with 10μl 2.5mM 4-hydroxytamoxifen (Sigma #H7904) in PBS or with the vehicle control ethanol in PBS at 2.5 dpa (approx. 36 hpa). The fish were heat shocked for 1h at 37°C at 66, 84 and 108 hpa. Fins were imaged at 3 dpa (approx. 72 hpa), 4 dpa (approx. 96 hpa) and 5 dpa (approx. 120 hpa) and analyzed for recombination events.

#### Electron microscopy

Fin regenerates were cut off distal to the amputation site and were fixed in 4% PFA in 100 mM phosphate buffer, pH 7.4. Samples were further dissected for embedding into epoxy resin and processed according to a modified protocol for serial block face SEM^67^ using osmium tetroxide (OsO_4_), thiocarbohydrazide (TCH), and again OsO_4_ to generate enhanced membrane contrast.^68^^,^^69^ In brief, samples were postfixed overnight in modified Karnovsky fixative (2% glutaraldehyde/2% formaldehyde in 50 mM HEPES, pH 7.4), followed by post-fixation in a 2% aqueous OsO_4_ solution containing 1.5% potassium ferrocyanide and 2mM CaCl_2_ (30 minutes on ice), washes in water, 1% TCH in water (20 minutes at room temperature), washes in water and a second osmium contrasting step in 2% OsO_4_/water (30 minutes on ice). Samples were washed and *en-bloc* contrasted with 1% uranyl acetate/water for 2 h on ice, washed in water, and dehydrated in a graded series of ethanol/water mixtures (30%, 50%, 70%, 90%, 96%), followed by 3 changes in pure ethanol on molecular sieve. Samples were infiltrated into the epon substitute EMBed 812 (resin/ethanol mixtures: 1:3, 1:1, 3:1 for 1h each, followed by pure resin overnight and for 5hrs), embedded into flat embedding molds, and cured at 65°C overnight. Ultrathin sections (70 nm) were prepared with a Leica UC6 ultramicrotome (Leica Microsystems, Wetzlar, Germany) and a diamond knife (Diatome, Nidau, Switzerland), collected on formvar-coated slot grids, and stained with lead citrate^70^ and uranyl acetate. In total, eight regenerating fin rays from five regenerates were analyzed. From each ray, three grids with six to nine sections were prepared and imaged. Mounted sections were analyzed with a JEM 1400Plus transmission electron microscope (JEOL, Freising, Germany) at 80 kV and images were taken with a Ruby digital camera (JEOL).

#### Drug treatments

Fgf inhibition was performed with 17 μM SU5402 (Selleckchem) or the vehicle control dimethylsulfoxide (DMSO, final percentage 0.1 %) in fish water at the times indicated. Solutions were changed daily. 50 mg/ml 5-Bromo-2-deoxyuridine (BrdU) (Sigma-Aldrich #B5002) stock solutions were prepared in DMSO and used at 5 mM (Figures 5A–5F and 7A–7F) or 2.5 mM (Figures 6C–6H) in selected experiments, either in fish water, in fish water supplemented with NFP (Sigma-Aldrich) or DMSO (3 % DMSO maximum), or in fish water supplemented with SU5402 or DMSO. BrdU treatment was performed during the last 6 hours of day 5 post amputation by incubation. NTR mediated ablation was performed with 1 μM NFP in fish water. Fin regenerates were either fixed and/or photographed at 5 dpa or zebrafish were allowed to recover from treatment for 2 days before fin regenerates were fixed and/or photographed.

### Quantification and statistical analysis

#### Statistical analysis

Statistical analysis was performed using Prism 6 (GraphPad Software, La Jolla, CA, USA), with the statistical tests and corresponding p values reported in the figures and respective legends. All values represent the mean ± SD unless otherwise stated. Additional information is listed in Table S9.

#### Live imaging, quantification of fluorescent cells and plot profile measurements

For quantification of GFP expression fish were anesthetized with 0.02% Tricaine (MS222) and their fins were imaged with a Zeiss SteREO Discovery.V12 microscope equipped with a AxioCam MRm camera and AxioVison software version 4.7.1.0. Identical settings for magnification, exposure time, gain, and contrast were used. Live imaging of *siam*:mCherry x *runx2*:GFP and *siam*:mCherry x *osterix*:GFP transgenic zebrafish was performed on a Dragonfly Spinning Disk confocal equipped with an Andor Zyla PLUS monochrome sCMOS camera and Fusion software with a z interval of 2 μm and a 30x silicone objective. GFP and mCherry double+ cells were counted in 50 μm intervals beginning with the most distal mCherry signal (0 μm) up to 400 μm. Quantification of reporter fluorescence along fin rays of transgenic *siam*:mCherry x *osterix*:GFP zebrafish was done with the plot profile tool in Image J/Fiji.

## Supplemental information

Document S1. Figures S1‒S11 and Tables S9 and S10

Table S1. Comparison of differentially expressed genes between Reg1 and following fin regeneration rounds (Reg2-4), related to Figure 1

Table S2. Differentially expressed genes of the epidermal (Basal), bone-forming (Osteo) and (non-osteoblast) blastemal (Blastema) cell main clusters, related to Figure 1

Table S3. Gene ontology terms of the blastema cell (Blastema) main cluster, related to Figure 1

Table S4. Gene ontology terms of the osteoblast (Osteo) main cluster, related to Figure 1

Table S5. Gene ontology terms of the basal epidermal cell (Basal) main cluster, related to Figure 1

Table S6. Differentially expressed genes of the blastema cell subclusters (Blastema0, Blastema1, Blastema2, Blastema3), related to Figures 1 and 2

Table S7. Differentially expressed genes of the osteoblast subclusters (Osteo0, Osteo1, Osteo2, Osteo3, Osteo4), related to Figures 1 and 2

Table S8. Differentially expressed genes of the basal epidermal cell subclusters (Basal0, Basal1), related to Figure 1
