# Supplementary material for: Compartmentalization and synergy of osteoblasts drive bone formation in the regenerating fin
Source: iScience. 2024 Jan 8;27(2):108841. doi: 10.1016/j.isci.2024.108841 (PMC10838958; doi:10.1016/j.isci.2024.108841)
Supplement: Document S1. Figures S1‒S11 and Tables S9 and S10 [file mmc1.pdf]

iScience, Volume *27*

Supplemental information

Compartmentalization and synergy of osteoblasts drive bone formation in the regenerating fin

Nicole Cudak, Alejandra Cristina López-Delgado, Fabian Rost, Thomas Kurth, Mathias Lesche, Susanne Reinhardt, Andreas Dahl, Steffen Rulands, and Franziska Knopf

Supplemental information

A

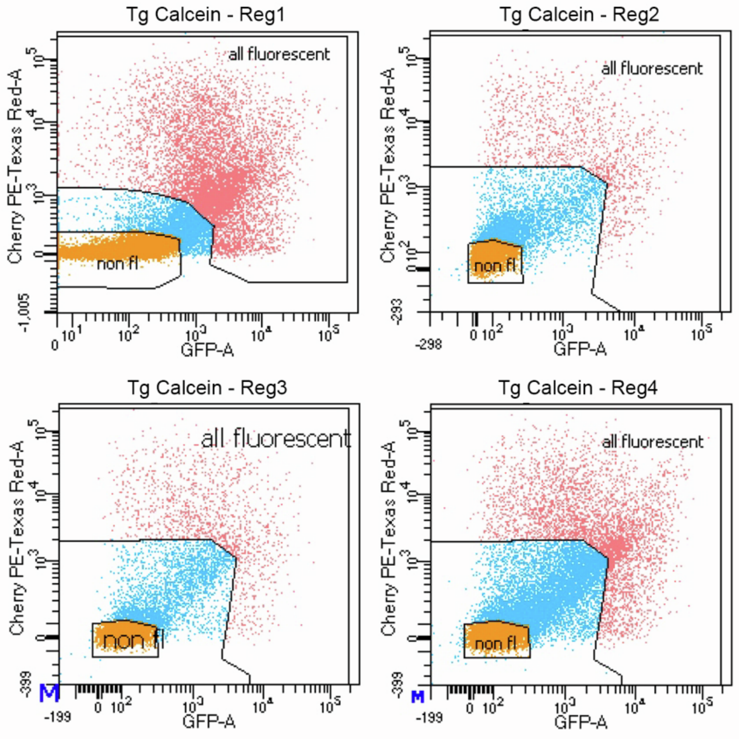

B

	Fraction of cells in %			
	**Reg1**	**Reg2**	**Reg3**	**Reg4**
**Calcein + live**	100	100	100	100
**mCherry+/GFP+/ mCherry+,GFP+**	30,7	13,8	16,4	20,8
**not fluorescent**	55,8	45,9	48,7	35,9
**not sorted cells**	13,5	40,3	34,9	43,3

C

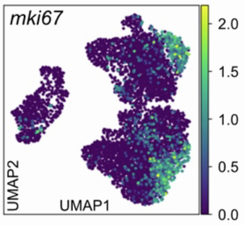

D

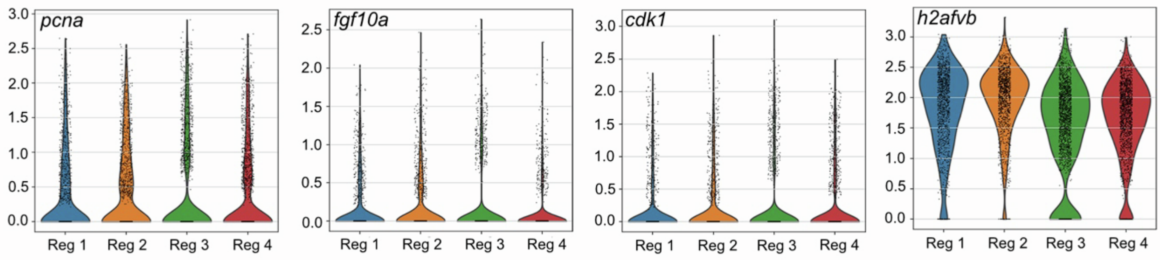

E

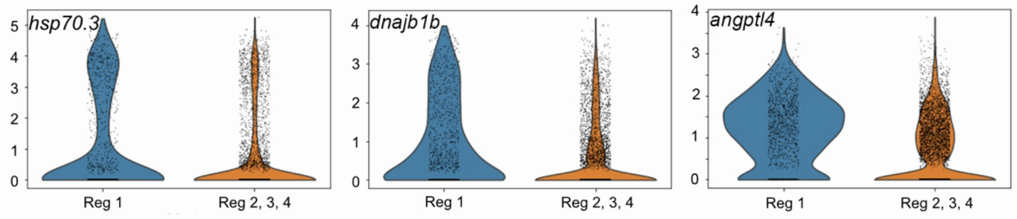

Figure S1. FACS conditions to isolate cells of interest, mki67 UMAP, and comparison of different regeneration experiments. Related to Figure 1. (A) Gatings for Reg1-Reg4. (B) Percentages of fluorescent (mCherry single+, GFP single+, mcherry/GFP double+), non-fluorescent and other non-sorted cells in Reg1-Reg4. (C) UMAP of the proliferation marker *mki67*. (D) Violin plots of *pcna*, *fgf10a*, *cdk1*, and *h2afvb* in different regeneration experiments. (E) Violin plots of *hsp70.3*, *dnajb1b*, and *angptl4* in Reg1 vs Reg2-4.

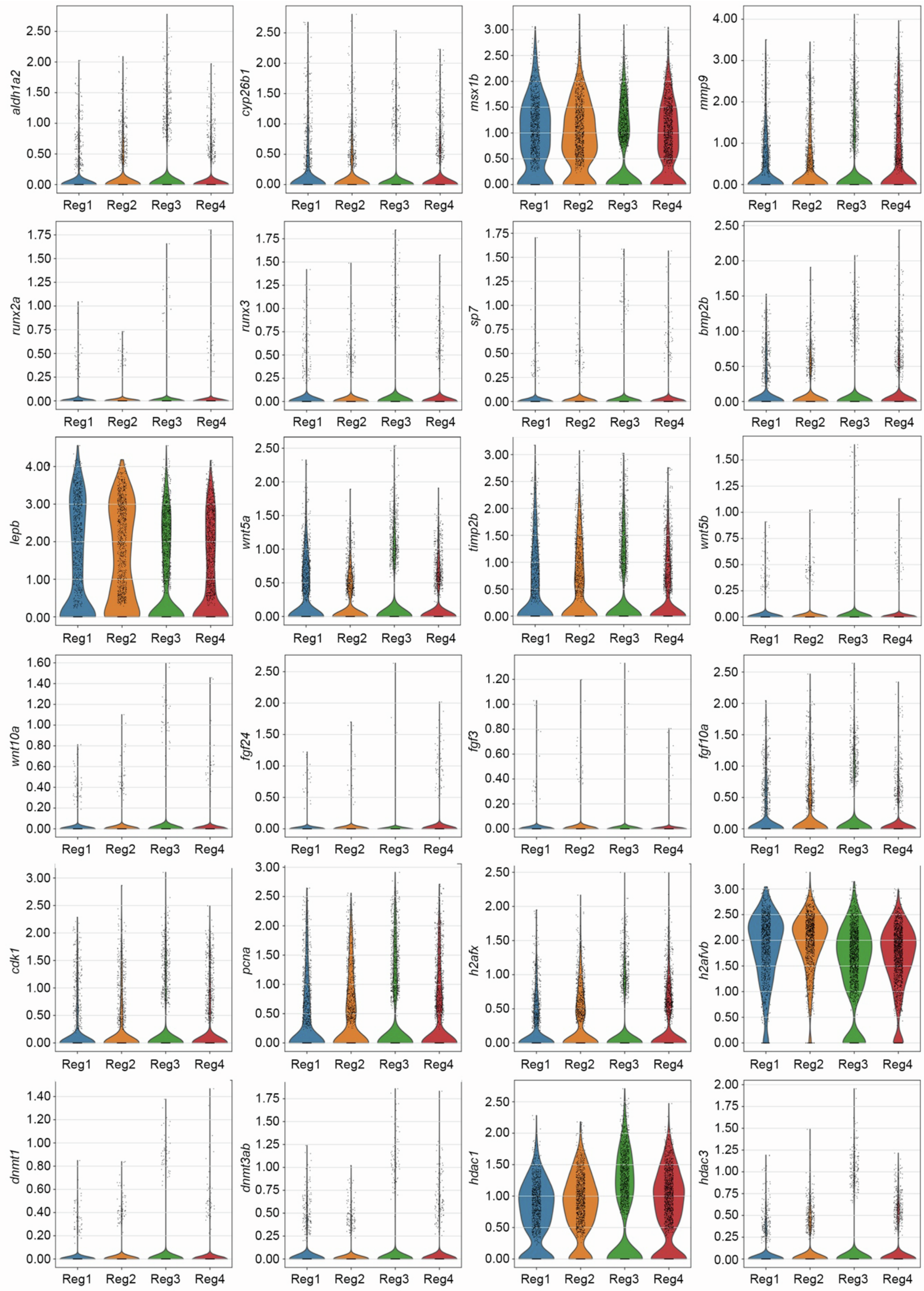

Figure S2. Selected gene expression in Reg1-Reg4 samples. Related to Figure 1. Neither a consistent boost or suppression of gene expression can be detected after repeated amputation. Violin plots of *aldh1a2, cyp26b1, msx1b, mmp9, runx2a, runx3, sp7, bmp2b, lepb, wnt5a, timp2b, wnt5b, wnt10a, fgf24, fgf3, fgf10a, cdk1, pcna, h2afx, h2afvb, dnmt1, dnmt3ab, hdac1, hdac3* in Reg1-4.

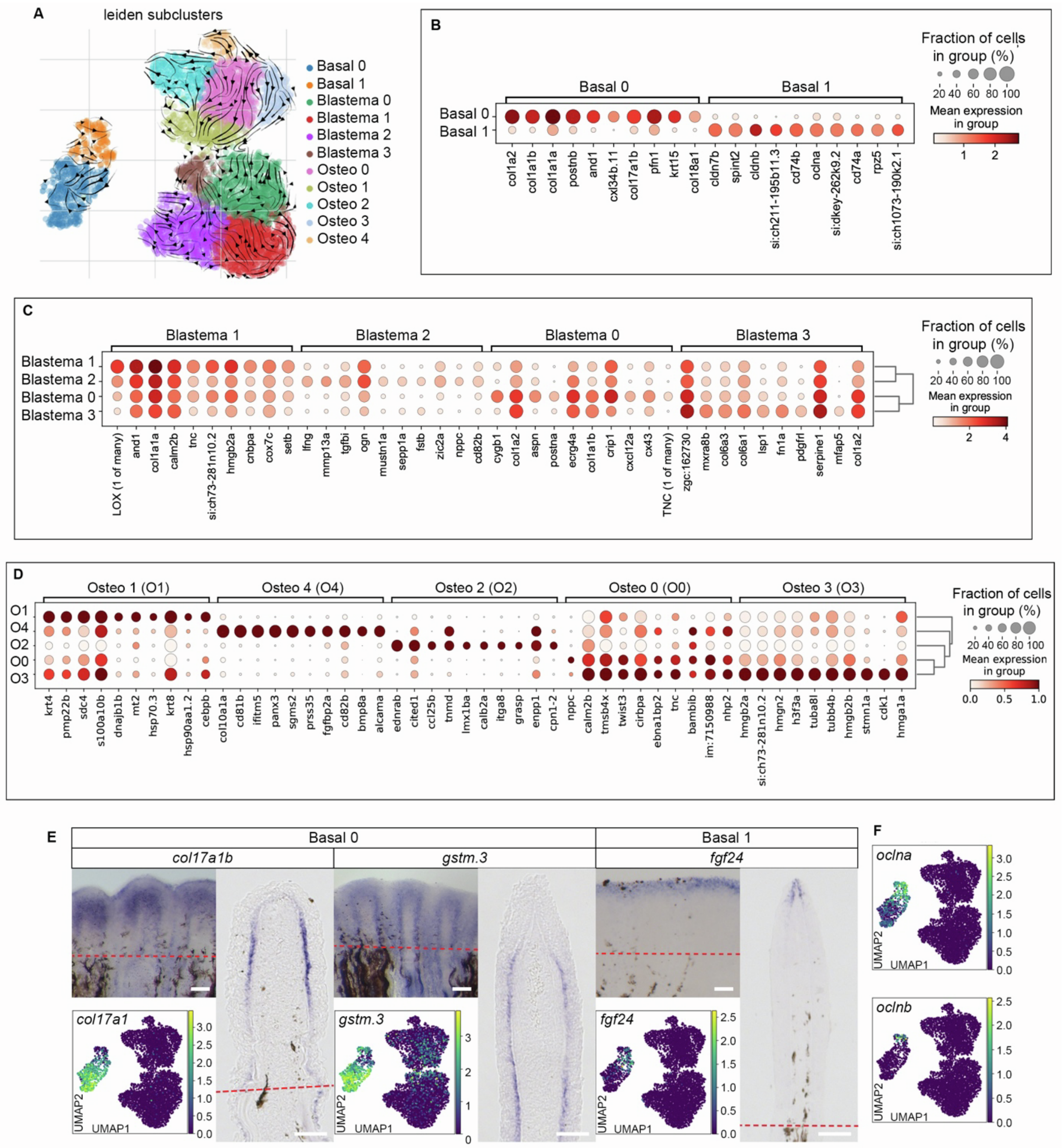

Figure S3. Gene expression in subclusters and discrimination of Basal0 and Basal1 cell clusters. Related to Figure 1 and 2. (A) RNA velocity plot (using the separate quantification of spliced and unspliced transcripts to infer the direction of gene expression regulation) not showing a clear pattern, possibly due to limitations of this method, such as the presence of multiple kinetic regimes or transcriptional boosts [1]. Given the uncertainties in RNA velocity analysis, we preferred PAGA analysis (Figure 1E) over RNA velocity analysis. (B) Gene expression in Basal subclusters. (C) Gene expression in Blastema subclusters. (D) Gene expression in Osteo subclusters. (E) Whole mount RNA *in situ* hybridization view, UMAP and cryosection view of *col17a1b*, *gstm.3* and *fgf24* expression. (F) UMAP views of *occludin a (oclna)* and *occludin b (oclnb)* expression.

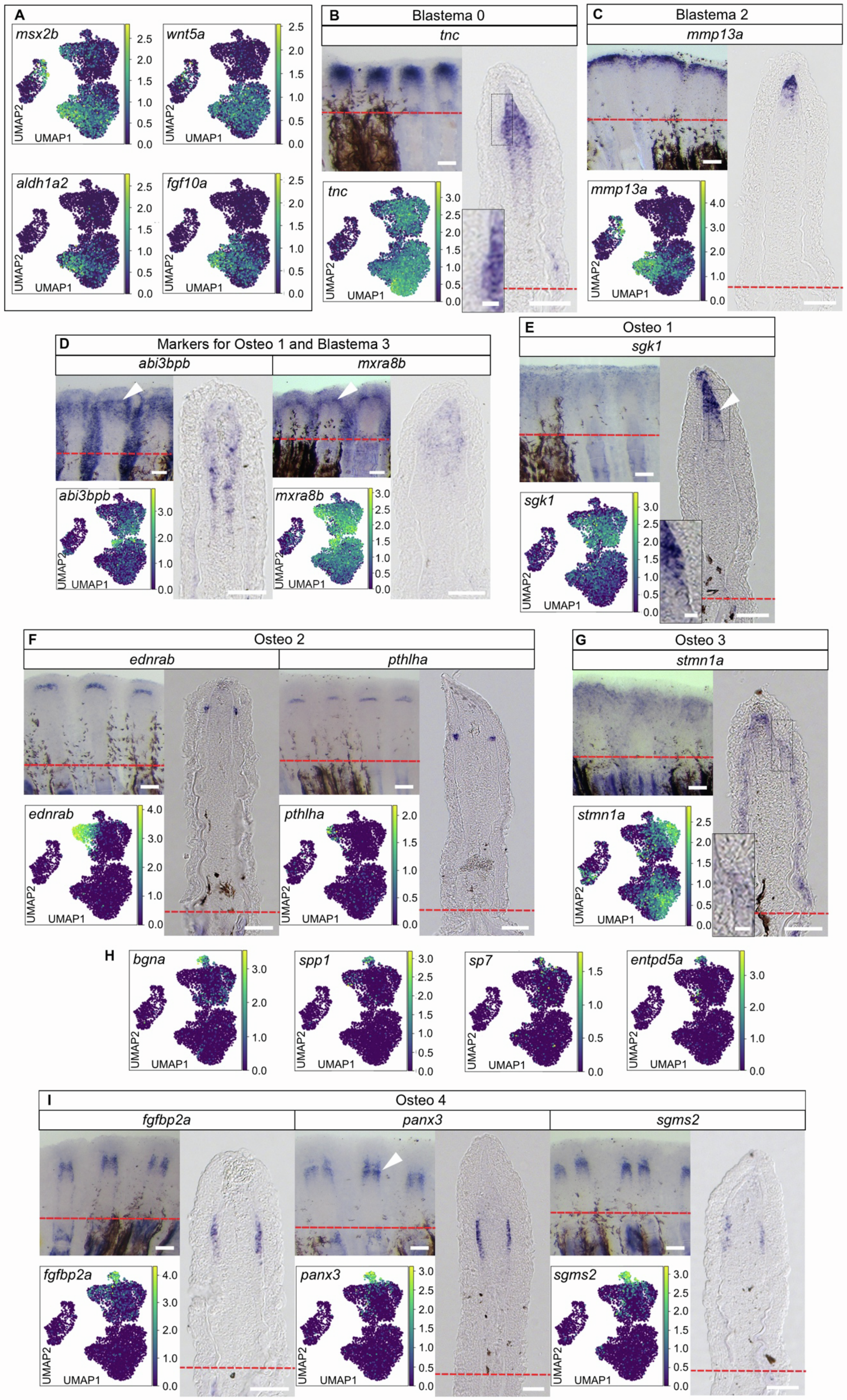
A
msx2b
wnt5a
aldh1a2
fgf10a
UMAP1
UMAP2
B
Blastema 0
tnc
C
Blastema 2
mmp13a
D
Markers for Osteo 1 and Blastema 3
abi3bpb
mxra8b
E
Osteo 1
sgk1
F
Osteo 2
ednrab
pthlha
G
Osteo 3
stmn1a
H
bgna
spp1
sp7
entpd5a
I
Osteo 4
fgfbp2a
panx3
sgms2

Figure S4. Markers of Blastema and Osteo clusters. Related to Figure 2. (A) Expression of known blastema markers *msx2b, wnt5a, aldh1a2, fgf10a* (UMAP views). (B) *tnc* expression in Blastema0. (C) *mmp13a* expression. (D) *abi3bpb* and *mxra8b* expression in Osteo1 and Blastema3 cells. (E) Non-exclusive *sgk1* expression in Osteo1. (F) *ednrab* and *pthlha* expression. (G) Non-exclusive *stmn1a* expression in Osteo3. (H) UMAPs of *bgna*, *spp1*, *sp7* and *entpd5* expression enriched in Osteo4. (I) *fgfbp2a*, *panx3* and *sgms2* expression. (A)-(G), (I) UMAP, WMISH and cryosection views. Scale bars whole mounts 100 µm, cryosections 50 µm, insets 10 µm.

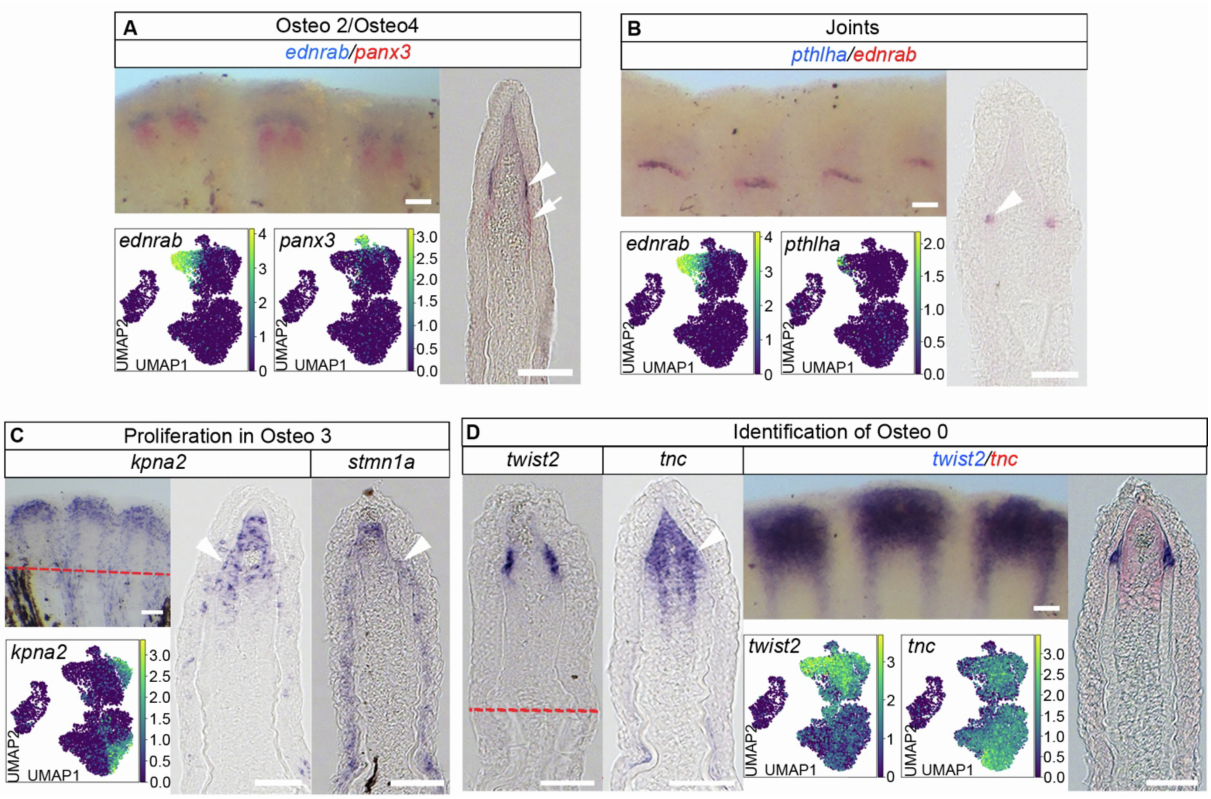

Figure S5. Characterization of specific osteoblast clusters. Related to Figure 2. (A) Distinct locations of Osteo2 (*ednrab*+, arrowhead) and Osteo4 cells (*panx3*+, arrow). Double ISH. (B) Co-labeling of *pthlha* and *ednrab* in Osteo2 cells (arrowhead). Double ISH. (C) Non-exclusive *kpna2* and *stmn1a* expression in Osteo3 (arrowheads). (D) Co-labeling of *tnc* and *twist2* in Osteo0 cells. Individual and double ISH. (A)-(D) UMAP, WMISH and cryosection views. Scale bars whole mounts 100 µm, cryosections 50 µm, insets 10 µm.

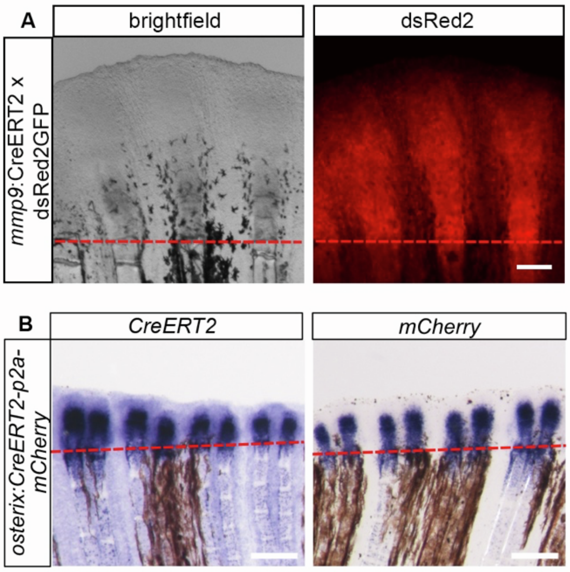

Figure S6. Expression of dsRed2 in *mmp9*:CreERT2 x *hsp70l*:R2nlsGFP and expression of *CreERT2* and *mCherry* in transgenic *osterix*:CreERT2-p2a-mCherry fins. Related to Figure 3. (A) Strong dsRed2 signal in 4 dpa regenerating fin rays after heat shock. Scale bar 200 µm. (B) Expression of *CreERT2* and *mCherry* is broad at 3 dpa. Whole mount ISH. Scale bar 100 µm.

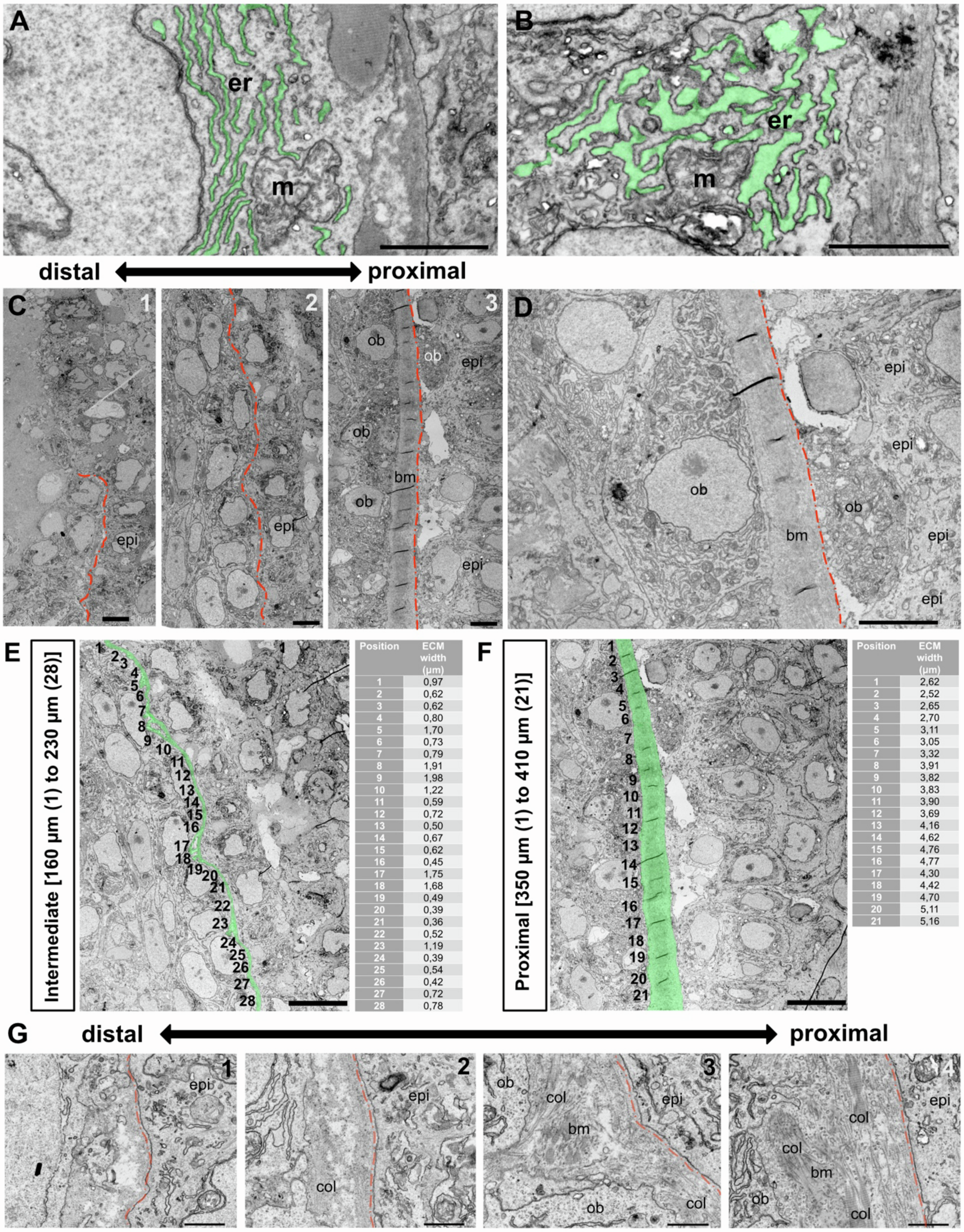

Position	ECM width (µm)
1	0,97
2	0,62
3	0,62
4	0,80
5	1,70
6	0,73
7	0,79
8	1,91
9	1,98
10	1,22
11	0,59
12	0,72
13	0,50
14	0,67
15	0,62
16	0,45
17	1,75
18	1,68
19	0,49
20	0,39
21	0,36
22	0,52
23	1,19
24	0,39
25	0,54
26	0,42
27	0,72
28	0,78

Position	ECM width (µm)
1	2,62
2	2,52
3	2,65
4	2,70
5	3,11
6	3,05
7	3,32
8	3,91
9	3,82
10	3,83
11	3,90
12	3,69
13	4,16
14	4,62
15	4,76
16	4,77
17	4,30
18	4,42
19	4,70
20	5,11
21	5,16

Figure S7. TEM of osteoblasts underlying the BLWE. Related to Figure 4. (A) Close-up of endoplasmatic reticulum (er, pseudo-colored in green) region shown in Figure 4B (position 2). (B) Close-up of er region (pseudo-colored in green) shown in Figure 4B (position 4). (C) Sequential overview images from distal (1) via intermediate (2) to proximal (3) regenerate regions. Red dashed lines, subepithelial basal lamina. epi, wound epidermis. Osteoblasts (ob) in more proximal regions begin to produce a thicker ECM layer (bone matrix, bm). (D) ob in C3 at higher magnification. Note the ob beyond the ECM/bm layer below the epi. (E) & (F) Thickness of the ECM (pseudo-colored in green) between epi and WE measured at different distances from the apical tip (measurements in displayed tables). (E) intermediate region (see

C2): 160 μm to 230 μm, (F) proximal region (see C3): 350 μm to 410 μm. (G) Images highlighting the ECM layer between epi and ob precursors/osteoblasts from distal to proximal. ECM thickness with collagen fibers (col) and electron dense bm material is more pronounced proximally (3,4). Scale bar (A) & (B) 2 μm, (C) & (D) 5 μm, (E) & (F) 10 μm, (G) 1 μm.

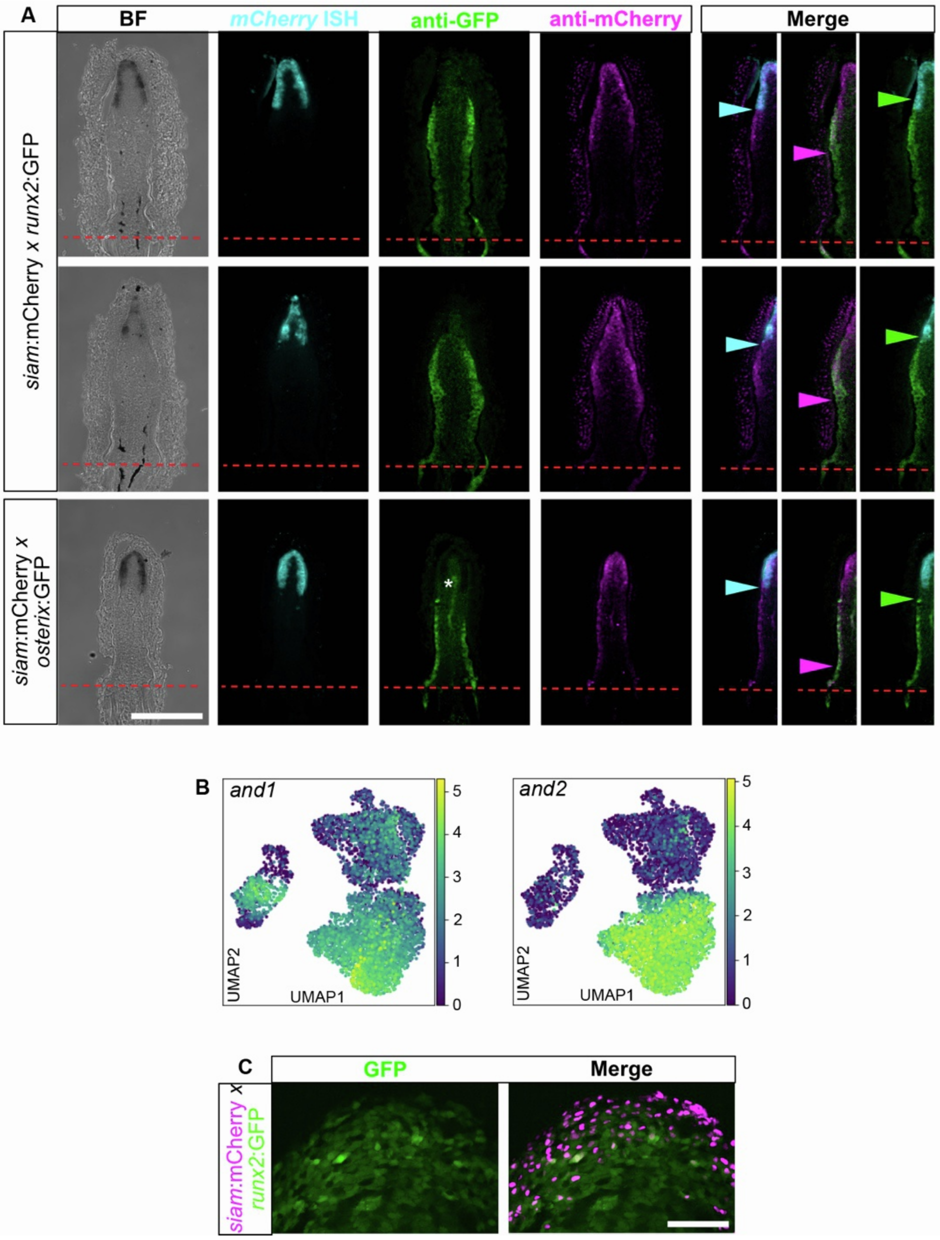

Figure S8. Expression ranges of distal blastema and osteoblast markers differ at the mRNA and protein level. Related to Figure 4. (A) Combined ISH and immunohistochemistry against *mCherry* mRNA, mCherry protein and GFP protein in transgenic *siam*:mCherry x *runx2*:GFP 3 dpa fin regenerates. The *mCherry* mRNA domain is much more restricted than the mCherry protein domain suggesting mixture of mCherry/GFP protein+ cells after transcription of *mCherry* has stopped. Asterisk, autofluorescent endothelial cells, cyan and green arrowheads, proximal limits of *mCherry* RNA and mCherry protein, respectively, magenta arrowhead, distal limit of GFP protein. Scale bar 100 μm. (B) UMAPs of *and1* and *and2* showing broad expression in the blastema. (C) Weak GFP protein expression is detectable in the distal blastema of transgenic *siam*:mCherry x *runx2*:GFP zebrafish at 3 dpa. Scale bar 100 μm.

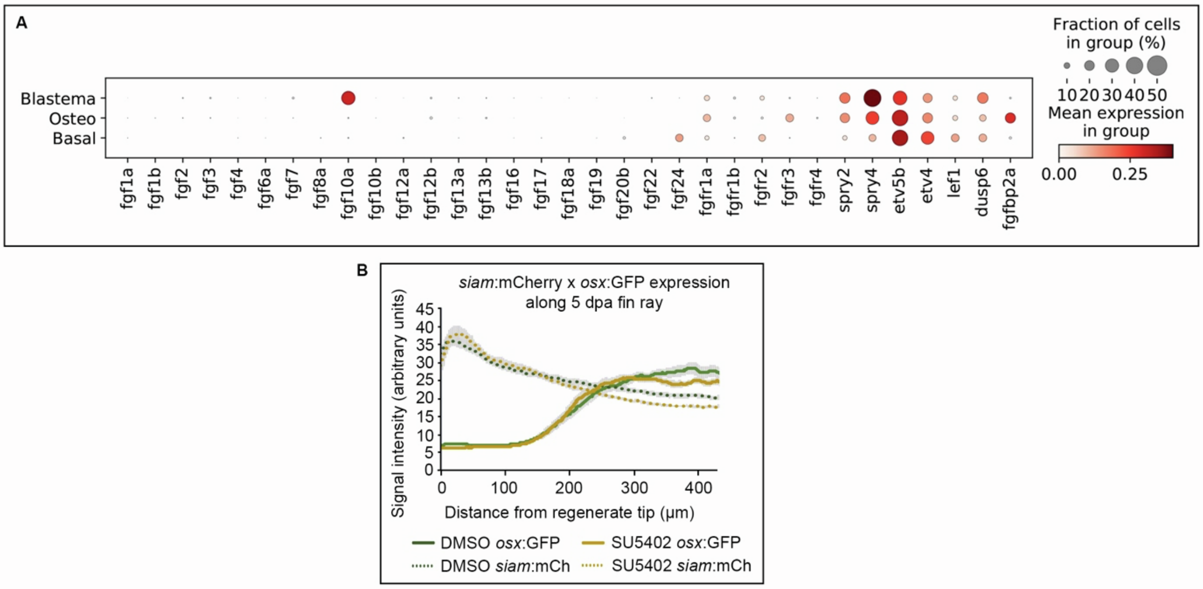

Figure S9. Fgf signaling pathway component expression and Fgfr1 inhibition. Related to Figure 5. (A) Expression of Fgf signaling components in the clusters Blastema, Osteo and Basal. (B) Fluorescence signal intensity of transgenic reporters (*osterix*:GFP, *siam*:mCherry) along the fin regenerate at 5 dpa, treated with DMSO or SU5402 from 3 to 5 dpa. Mean ± SEM.

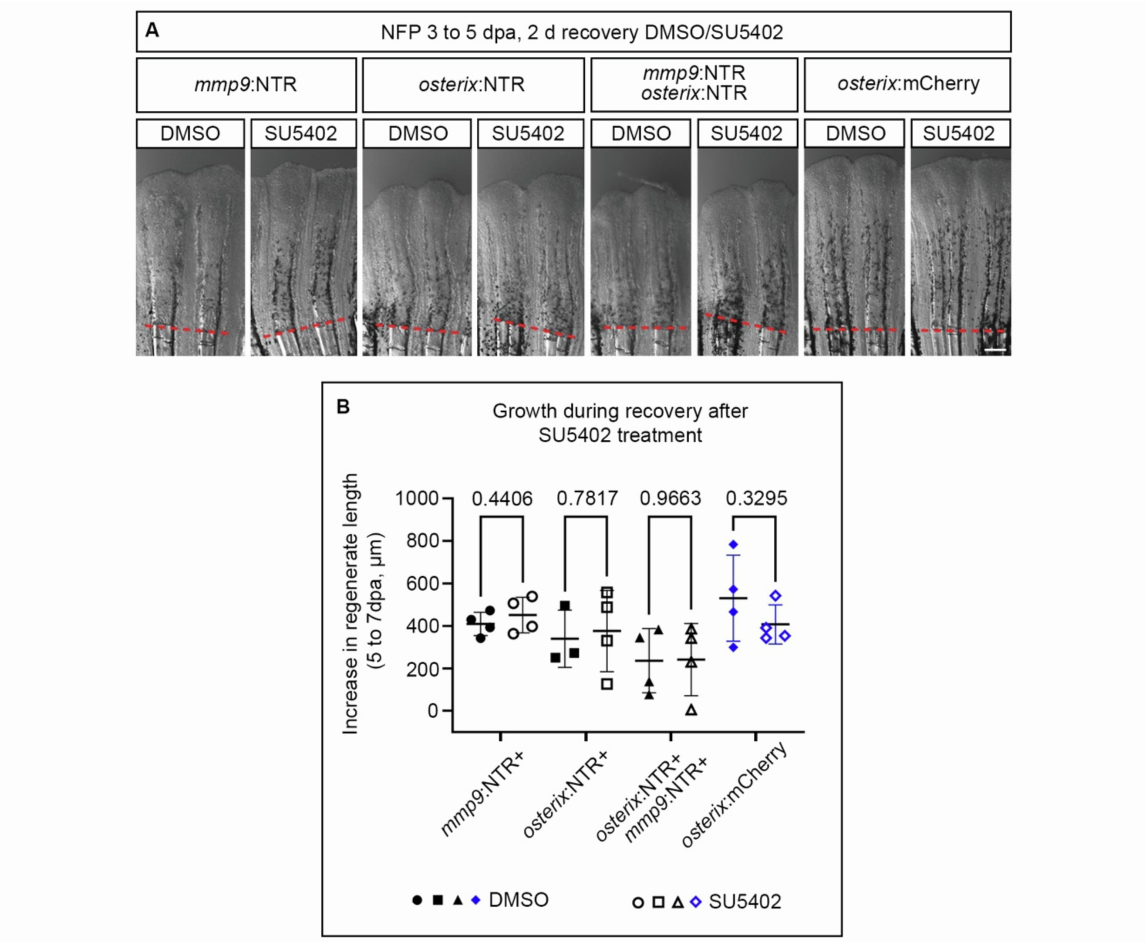

Figure S10. Regenerate recovery is not affected by Fgf inhibition. Related to Figure 6. (A) Representative images of fin regenerates treated either with DMSO or SU5402 during recovery after ablation. Scale bar 200 µm. (B) Quantification of increase of regenerate length during recovery period [experiment shown in (A)]. Welch's t-tests. Mean ± SD.

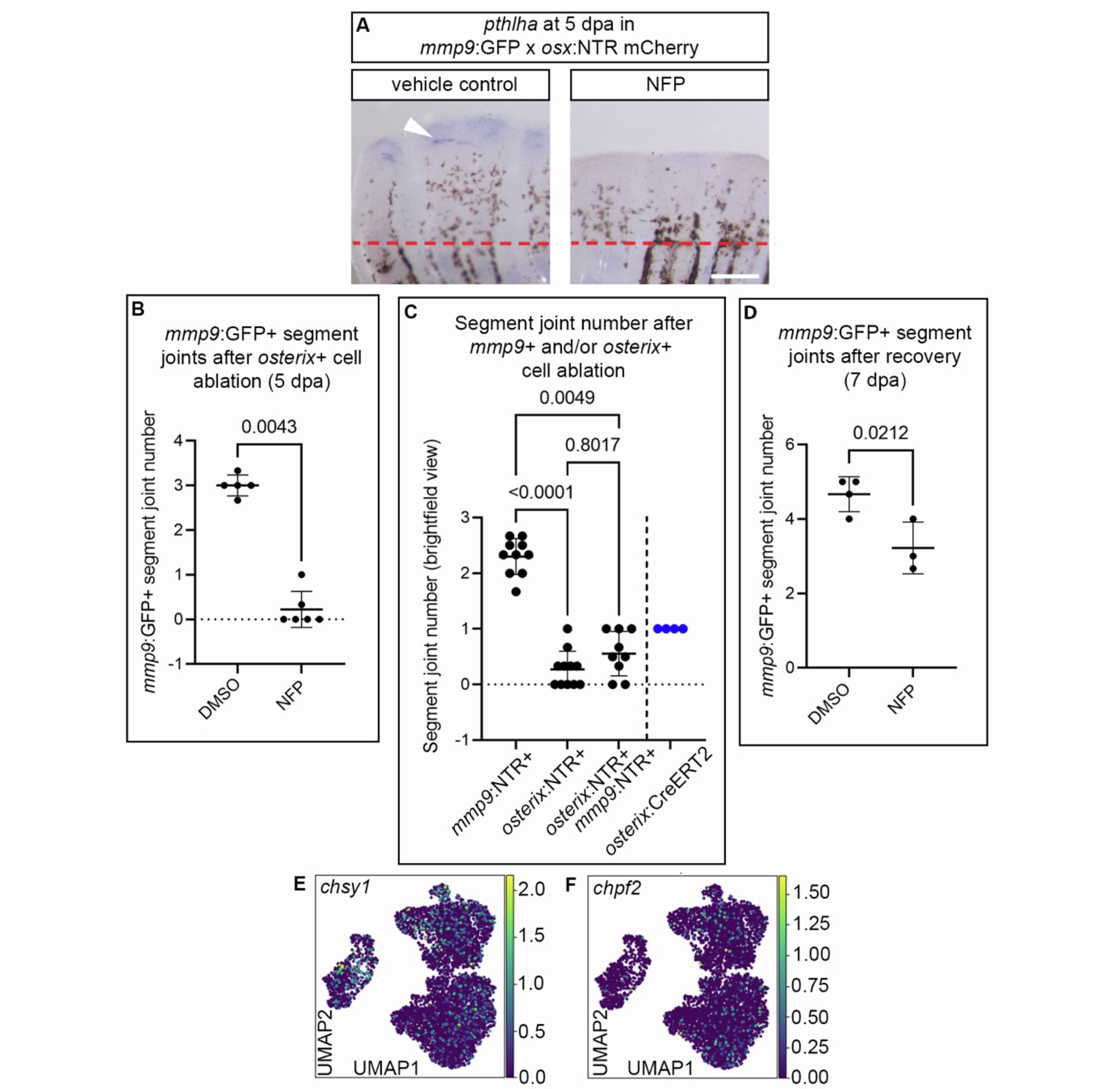

Figure S11. Altered segment joint formation upon *osterix*+ cell ablation. Related to Figure 6 and 7. (A) *pthlha* expression in vehicle control (DMSO) treated vs NFP treated *mmp9*:GFP x *osterix*:NTR mCherry treated fin regenerates. Arrowhead pointing at expression at prospective joint forming sites. Scale bar 200 µm. (B) Quantification of *mmp9*:GFP+ segment joint number in vehicle control (DMSO) treated vs NFP *mmp9*:GFP x *osterix*:NTR mCherry treated fin regenerates at 5 dpa (experiment shown in Figure 7D). Mann-Whitney. (C) Quantification of segment joints visible in the brightfield channel at 5 dpa after either *mmp9*:NTR+ cell ablation, *osterix*:NTR+ cell ablation, combined *mmp9*:NTR+ and *osterix*:NTR+ cell ablation, or control NFP treatment in (non-sibling) *osterix*:CreERT2 zebrafish (experiment shown in Figure 6A). Kruskal-Wallis. (D) Quantification of *mmp9*:GFP+ segment joint number in vehicle control (DMSO) treated vs NFP *mmp9*:GFP x *osterix*:NTR mCherry treated fin regenerates after recovery at 7 dpa (experiment shown in Figure 7E). Unpaired two-tailed t-test. (B)-(D) Average segment numbers calculated from 2-3 dorsal fin rays per individual. (E) UMAP of *chsy1*. (F) UMAP of *chpf2*. (B), (C), (D) Mean ± SD.

Table S9. Number of specimens used in experiments. Related to Figure 1-7. For whole mount RNA ISH: first number = number of specimens displaying the presented staining pattern, second number = total number of specimens used, e.g. 4/5 = 4 out of 5 specimens display the depicted expression pattern.

**Figure**	**Specimen number (n)**
**Figure 1**	**(A)** n (*mCherry/gfp*) = 9 cryosections.
**Figure 2**	**(A)** n (*zic2a*) = 3/3 fins (whole mount), n (*zic2a*) = 7 cryosections. n (*timp2b*) = 5/5 fins (whole mount), n (*timp2b*) = 10 cryosections. **(B)** n (*postna*) = 6/6 fins (whole mount), n (*postna*) = 20 cryosections. **(C)** n [*LOX (1 of many)*] = 3/4 fins (whole mount), n = 19 cryosections. **(D)** n (*mustn1a*) = 5/5 fins (whole mount), n (*mustn1a*) = 26 cryosections. **(E)** n (*mfap5*) = 5/5 fins (whole mount), n = 28 cryosections. **(F)** n (*fgl1*) = 5/5 fins (whole mount), n (*fgl1*) = 14 cryosections. n (*spon1b*) = 6/6 fins (whole mount), n (*spon1b*) = 19 cryosections. **(G)** n (*twist2*) = 4/5 fins (whole mount), n = 20 cryosections. **(H)** n (*lum*) = 5/5 fins (whole mount), n (*lum*) = 30 cryosections. **(I)** n (*ifitm5*) = 4/5 fins (whole mount), n (*ifitm5*) = 12 cryosections.
**Figure 3**	**(B)-(D)** n (EtOH) = 7 fish, n (4-OHT) = 8 fish. **(E) & (G) & (H)** n (EtOH) = 8 fish, n (4-OHT) = 5 fish. **(I)** n = 3 fish.
**Figure 4**	**(A) & (B)** n = 5 fish with 8 fin rays. **(E)** n (*mCherry/gfp*) = 10/10 fins (whole mount), n (*mCherry/gfp*) = 19 cryosections. n (*mCherry*) = 109 cryosections, n (*gfp*) = 51 cryosections. **(F)** n = 11 rays of 5 different fish. **(G)** n = 11 rays (*siam*:mCherry x *runx2*:GFP) or 10 rays (*siam*:mCherry x *osterix*:GFP) of 5 different fish. **(H)** n = 4/4 fins (whole mount), n = 49 cryosections. **(I)** n = 10 rays of 5 different fish.
**Figure 5**	**(B) & (C)** n (DMSO/SU5402) = 10. **(D)-(F)** n (DMSO/SU5402) = 6.
**Figure 6**	**(A) & (B)** n (*mmp9*:NTR+) = 10, n (*osterix*:NTR+) = 11, n (*osterix*:NTR+, *mmp9*:NTR+) = 9 n (*osterix*:CreERT2+) = 4. **(C) & (D)** n (*osterix*:mCherry) = 8 specimen with 17 sections, n (*osterix*:NTR+, *mmp9*:NTR+) = 7 with 12 sections. **(F)** n (*osterix*:NTR+, *mmp9*:NTR+, 5 dpa) = 8, n (*osterix*:NTR+, *mmp9*:NTR+, 7 dpa) = 4. **(G)** n (all groups except *osterix*:NTR+ DMSO) = 4, n (*osterix*:NTR+ DMSO) = 3. **(H)** n (all groups) = 4.
**Figure 7**	**(A) & (C)** n (*runx2*:GFP, *osterix*:NTR-, NFP) = 6, n (*runx2*:GFP, *osterix*:NTR+, NFP) = 9. **(B)** n (*runx2*:GFP, *osterix*:NTR-, NFP) = 5, n (*runx2*:GFP, *osterix*:NTR+, NFP) = 9. **(D)** n (vehicle control/DMSO) = 5, n (NFP) = 6. **(E)** n (vehicle control/DMSO) = 4, n (NFP) = 3. **(F)** n (*runx2*:GFP, *osterix*:NTR-, NFP) = 3 with 96 sections, n (*runx2*:GFP, *osterix*:NTR+, NFP) = 4 with 63 sections.
**Figure S3**	**(D)** n (*col17a1*) = 6/6 fins (whole mount), n (*col17a1*) = 25 cryosections. n (*gstm3*) = 4/4 fins (whole mount), n (*gstm3*) = 21 cryosections. n (*fgf24*) = 4/4 fins (whole mount), n (*fgf24*) = 24 cryosections

**Figure S4**	**(B)** n (*tnc*) = 5/5 fins (whole mount), n (*tnc*) = 11 cryosections. **(C)** n (*mmp13a*) = 5/5 fins (whole mount), n (*mmp13a*) = 33 cryosections. **(D)** n (*abi3bpb*) = 5/5 fins (whole mount), n (*abi3bpb*) = 35 cryosections. n (*mxra8b*) = 5/5 fins (whole mount), n (*mxra8b*) = 13 cryosections. **(E)** n (*sgk1*) = 4/5 fins (whole mount), n (*sgk1*) = 10 cryosections. **(F)** n (*ednrab*) = 4/5 fins (whole mount), n (*ednrab*) = 9 cryosections. n (*pthlha*) = 5/5 fins (whole mount), n (*pthlha*) = 7 cryosections. **(G)** n (*stmn1a*) = 5/5 fins (whole mount), n = 9 cryosections. **(I)** n (*fgfbp2a*) = 5/5 fins (whole mount), n (*fgfbp2a*) = 6 cryosections. n (*panx3*) = 5/5 fins (whole mount), n (*panx3*) = 8 cryosections. n (*sgms2*) = 5/5 fins (whole mount), n (*sgms2*) = 12 cryosections.
**Figure S5**	**(A)** n (*ednrab/panx3*) = 3/3 fins (whole mount), n = 12 cryosections. **(B)** n (*pthlha/ednrab*) = 5/5 fins (whole mount), n = 11 cryosections. **(C)** n (*kpna2*) = 4/5 fins (whole mount), n (*kpna2*) = 11 cryosections. n (*stmn1a in situ* hybridization) see Figure S3F. **(D)** n (*tnc/twist2*) = 5/5 fins (whole mount), n = 10 cryosections. n (individual *tnc* & *twist2 in situ* hybridizations) see Figure S4B, Figure 2G.
**Figure S6**	n (*CreERT2*) = 5/5 fins, n (*mCherry*) = 5/5 fins.
**Figure S7**	**(A)-(G)** n = 5 fins with 8 fin rays.
**Figure S8**	**(A)** n (*siam:*mCherry *x runx2:*GFP) = 4 fish with 6 cryosections. n (*siam:*mCherry *x osterix:*GFP) = 3 fish with 3 cryosections. (C) n (*siam:*mCherry *x runx2:*GFP) = 5 fish with 1-2 rays per fish.
**Figure S9**	**(B)** n (DMSO/SU5402) = 10
**Figure S10**	**(A) & (B)** n (all groups except *osterix*:NTR+ DMSO) = 4, n (*osterix*:NTR+ DMSO) = 3.
**Figure S11**	**(A)** n (DMSO) = 5, n (NFP) = 4. **(B)** n (vehicle control/DMSO) = 5, n (NFP) = 6. **(C)** n (*mmp9*:NTR+) = 10, n (*osterix*:NTR+) = 11, n (*mmp9*:NTR+, *osterix*:NTR+) = 9, n (*osterix*:CreERT2+) = 4. **(D)** n (vehicle control/DMSO) = 4, n (NFP) = 3.

Table S10. Full gene names in alphabetical order. Related to Figure 1-7.

**Gene symbol**	**Gene full name**
abi3bpb	*ABI family, member 3 (NESH) binding protein b*
aldh1a2	*aldehyde dehydrogenase 1 family, member A2*
and1	*actinodin1*
and2	*actinodin2*
bgna	*biglycan a*
bmp4	*bone morphogenetic protein 4*
cdh4	*cadherin 4*
cdh11	*cadherin 11*
chpf2	*chondroitin polymerizing factor 2*
chsy1	*chondroitin sulfate synthase 1*
cldni	*claudin i*
Col1a2	*Collagen, type X, alpha 2*
col10a1a	*collagen, type X, alpha 1a*

col17a1b	*collagen, type XVII, alpha 1b*
crip2	*cysteine-rich protein 2*
cx43 (=gja1b)	*gap junction protein alpha 1b*
dnajb1b	*DnaJ heat shock protein family (Hsp40) member B1b*
dkk1a	*dickkopf WNT signaling pathway inhibitor 1a*
dkk1b	*dickkopf WNT signaling pathway inhibitor 1b*
dlx5a	*distal-less homeobox 5a*
ednrab	*endothelin receptor type Ab*
entpd5a	*ectonucleoside triphosphate diphosphohydrolase 5a*
epcam	*epithelial cell adhesion molecule*
evx1	*even-skipped homeobox 1*
fgf3	*fibroblast growth factor 3*
fgf10a	*fibroblast growth factor 10a*
fgf24	*fibroblast growth factor 24*
fgfbp2	*fibroblast growth factor binding protein 2a*
fgl1	*fibrinogen-like 1*
fhl1a	*four and a half LIM domains 1a*
fn1b	*fibronectin 1b*
gstm.3	*glutathione S-transferase mu tandem duplicate 3*
her6	*hairy-related 6*
hoxa13a	*homeobox A13a*
hsp70.3	*heat shock cognate 70-kd protein, tandem duplicate 3,*
ifitm5	*interferon induced transmembrane protein 5*
igf2b	*insulin-like growth factor 2b*
junba	*JunB proto-oncogene, AP-1 transcription factor subunit a*
junbb	*JunB proto-oncogene, AP-1 transcription factor subunit b*
kpna2	*karyopherin alpha 2 (RAG cohort 1, importin alpha 1)*
krt5	*keratin 5*
lamb1a	*laminin, beta 1a*
lef1	*lymphoid enhancer-binding factor 1*
lepb	*leptin b*
lfng	*LFNG O-fucosylpeptide 3-beta-N-acetylglucosaminyltransferase*
LOX	*lysyl oxidase*
lum	*lumican*
mfap5	*microfibril associated protein 5*
mki67	*marker of proliferation Ki-67*
mmp13a	*matrix metallopeptidase 13a*
mmp 9	*matrix metallopeptidase 9*
msx2b	*muscle segment homeobox 2b*
msx3	*muscle segment homeobox 3*
mustn1a	*musculoskeletal, embryonic nuclear protein 1a*
mxra8b	*matrix-remodelling associated 8b*
oclna	*occludin a*

oclnb	*occludin b*
osteocalcin	*bone gamma-carboxyglutamate (gla) protein*
panx3	*pannexin 3*
pcna	*proliferating cell nuclear antigen*
pdgfrl	*platelet-derived growth factor receptor-like*
phlda2	*pleckstrin homology-like domain, family A, member 2*
postna	*periostin, osteoblast specific factor a*
Prrx1a	*Paired related homeobox 1a*
pthlha	*parathyroid hormone-like hormone a*
RUNX2	*RUNX Family Transcription Factor 2*
sgk1	*serum/glucocorticoid regulated kinase 1*
sgm2	*sphingomyelin synthase 2a*
shha	*sonic hedgehog a*
siam	*siamois*
sp7	*Sp7 transcription factor, osterix, osx*
spon1b	*spondin 1b*
spp1	*secreted phosphoprotein 1*
spry4	*sprouty homolog 4*
stmn1a	*stathmin 1a*
tgfbi	*transforming growth factor, beta-induced*
timp2b	*TIMP metallopeptidase inhibitor 2b*
tnc	*tenascin Cb*
wnt3a	*wingless-type MMTV integration site family, member 3A*
wnt5b	*wingless-type MMTV integration site family, member 5b*
zic2a	*zic family member 2 (odd-paired homolog, Drosophila), a*

References

1. Bergen, V., Soldatov, R.A., Kharchenko, P. V, and Theis, F.J. (2021). RNA velocity—current challenges and future perspectives. Mol. Syst. Biol. *17*, e10282. https://doi.org/10.15252/msb.202110282.
